# Visualization of endogenous p27 and Ki67 reveals the importance of a c-Myc-driven metabolic switch in promoting survival of quiescent cancer cells

**DOI:** 10.7150/thno.63763

**Published:** 2021-09-21

**Authors:** Ting La, Song Chen, Tao Guo, Xiao Hong Zhao, Liu Teng, Dandan Li, Michael Carnell, Yuan Yuan Zhang, Yu Chen Feng, Nicole Cole, Alexandra C. Brown, Didi Zhang, Qihan Dong, Jenny Y. Wang, Huixia Cao, Tao Liu, Rick F. Thorne, Feng-Min Shao, Xu Dong Zhang, Lei Jin

**Affiliations:** 1 School of Biomedical Sciences and Pharmacy, The University of Newcastle, NSW, 2308, Australia.; 2 Translational Research Institute, Henan Provincial People's Hospital and People's Hospital of Zhengzhou University, Henan Provincial and Zhengzhou City Key laboratory of Long Non-coding RNA and Cancer Metabolism, Henan, 450053, China.; 3 Centre for Excellence in Molecular Plant Sciences, Chinese Academy of Sciences, Shanghai, 200032, China.; 4 Department of Pulmonary and Critical Care Medicine, Henan Provincial People's Hospital, Zhengzhou University People's Hospital, Henan 450003, China.; 5 Biomedical Imaging Facility, University of New South Wales, NSW, 2052, Australia.; 6 Department of Orthopaedics, John Hunter Hospital, Hunter New England Health, NSW, 2305, Australia.; 7 Central Clinical School and Charles Perkins Centre, The University of Sydney, Sydney 2006, Australia.; 8 Children's Cancer Institute Australia for Medical Research, University of New South Wales, NSW 2750, Australia.; 9 Department of Nephrology, Henan Provincial People's Hospital, Zhengzhou University People's Hospital, Henan Provincial Clinical Research Canter for Kidney Disease, Henan 450003, China.

**Keywords:** c-Myc, IDH3, quiescence, quiescent cells, oxidative phosphorylation

## Abstract

**Rationale:** Recurrent and metastatic cancers often undergo a period of dormancy, which is closely associated with cellular quiescence, a state whereby cells exit the cell cycle and are reversibly arrested in G0 phase. Curative cancer treatment thus requires therapies that either sustain the dormant state of quiescent cancer cells, or preferentially, eliminate them. However, the mechanisms responsible for the survival of quiescent cancer cells remain obscure.

**Methods:** Dual genome-editing was carried out using a CRISPR/Cas9-based system to label endogenous p27 and Ki67 with the green and red fluorescent proteins EGFP and mCherry, respectively, in melanoma cells. Analysis of transcriptomes of isolated EGFP-p27^high^mCherry-Ki67^low^ quiescent cells was conducted at bulk and single cell levels using RNA-sequencing. The extracellular acidification rate and oxygen consumption rate were measured to define metabolic phenotypes. SiRNA and inducible shRNA knockdown, chromatin immunoprecipitation and luciferase reporter assays were employed to elucidate mechanisms of the metabolic switch in quiescent cells.

**Results:** Dual labelling of endogenous p27 and Ki67 with differentiable fluorescent probes allowed for visualization, isolation, and analysis of viable p27^high^Ki67^low^ quiescent cells. Paradoxically, the proto-oncoprotein c-Myc, which commonly drives malignant cell cycle progression, was expressed at relatively high levels in p27^high^Ki67^low^ quiescent cells and supported their survival through promoting mitochondrial oxidative phosphorylation (OXPHOS). In this context, c-Myc selectively transactivated genes encoding OXPHOS enzymes, including subunits of isocitric dehydrogenase 3 (IDH3), whereas its binding to cell cycle progression gene promoters was decreased in quiescent cells. Silencing of c-Myc or the catalytic subunit of IDH3, IDH3α, preferentially killed quiescent cells, recapitulating the effect of treatment with OXPHOS inhibitors.

**Conclusion:** These results establish a rigorous experimental system for investigating cellular quiescence, uncover the high selectivity of c-Myc in activating OXPHOS genes in quiescent cells, and propose OXPHOS targeting as a potential therapeutic avenue to counter cancer cells in quiescence.

## Introduction

Cellular quiescence refers to a state whereby cells exit the cell cycle and are reversibly arrested in G0 phase 1. It is a fundamental physiological mechanism ensuring tissue homeostasis and also impinges on many pathological conditions 2-4. In particular, quiescent cancer cells are resistant to anti-cancer therapeutics and underlie cancer recurrence and metastasis 5, 6. Curative cancer treatment thus requires maintaining such cells in a perpetual quiescent state or alternatively employing the means to eliminate them 5, 6. However, the mechanisms responsible for cancer cell quiescence remain to be fully understood, even though a number of cellular machineries, such as the MEK/ERK and p38 mitogen activated protein kinase pathways, p53 signalling and the unfolded protein response are known to participate in regulating cellular quiescence 7-10. This is closely related to technical difficulties associated with purifying and characterizing viable quiescent cells, as no specific markers are expressed on their surface that allow for differentiating them from cycling cells using conventional approaches 11, 12.

Quiescent cells characteristically express high levels of the cyclin-dependent kinase inhibitor p27 and low levels of the proliferation marker Ki67, two proteins primarily located to the nucleus 12, 13. Purification of viable quiescent cancer cells without permeabilization with these characteristics represents a promising yet challenging approach 14. Introduction of an exogenous fluorescence protein-labelled p27 mutant, which remains susceptible to degradation by the ubiquitin-proteasome system, has been introduced as a marker for identification of quiescent cells 15, whereas the expression of green fluorescence protein (GFP) driven by the promoter of *MKI67*, the gene encoding Ki67, was proposed as a method to distinguish cycling cells from quiescent cells 11. However, regulation of the cellular levels of these exogenous probes is limited to defined mechanisms 16, which is conceivably unable to echo the complexity of regulation of the expression of endogenous p27 and Ki67 by diverse biological processes 17-20.

We have developed a CRISPR/Cas9-based system that fuses sequences encoding enhanced GFP (EGFP) to the endogenous locus of *CDKN1B*, the gene encoding p27, and sequences encoding the red fluorescent protein mCherry to the endogenous locus of *MKI67*. Here we report that endogenous p27 and Ki67 labelled with differentiable fluorescent proteins are reliable markers allowing for unbiased, sensitive visualization and isolation of p27^high^Ki67^low^ quiescent cells. By using this system, we have found that c-Myc, which is important for malignant cell cycle progression 21, is paradoxically expressed at relatively high levels and drives mitochondrial OXPHOS in p27^high^Ki67^low^ quiescent melanoma cells. Mechanistically, c-Myc selectively activates transcription of genes encoding subunits of isocitric dehydrogenase (IDH3) and many other OXPHOS enzymes through increased occupancy of their promoters in melanoma cells in quiescence. Moreover, we show that inhibition of OXPHOS preferentially kills quiescent melanoma cells, with implications of OXPHOS targeting for overcoming resistance of quiescent cancer cells to treatment.

## Materials and Methods

### Cell culture

The human melanoma cell lines ME4405, IgR3, MM200, Mel-RM, and A375 and the Mel-RM and A375 sublines with dual genome-editing of endogenous p27 and Ki67 were cultured in DMEM containing 5% FCS 8. These cell lines were verified to be free of mycoplasma contamination every 3 months and were authenticated by short tandem repeat (STR) profiling by Australian Genome Research Facility (AGRF).

### Generation of Mel-RM and A375 sublines with dual genome-editing of endogenous p27 and Ki67

Single-guide (sg) RNAs were designed to target the sequences next to the stop codon of human *CDKN1B* and *MKI67* genes, respectively. Annealed oligonucleotides comprising the adaptor-target-adaptor sequence (Table S1) were constructed into the BsmBI-digested lentiCRISPR v2 (Addgene plasmid #52961) vector, which contains a SpCas9 expression cassette and a U6 promoter driving sgRNA expression. The donor DNA fragments carrying *EGFP* (flanked by a 1097 bp left arm and a 1256 bp right arm) and *mCherry* sequences (flanked by a 1288 bp left arm and a 920 bp right arm) (Figure S1), respectively, were synthesized (HuaGene Biotech Co., Ltd, Shanghai) and then inserted into pEASY Blunt-Zero vector (TransGen Biotech). *CDKN1B/EGFP* donor was linearized by PstI-HF (NEB, R3140L) and *MKI67/mCherry* donor was linearized by SpeI-HF (NEB, R3133L) before nucleofection (Figure S1). The sgRNA expression vector carrying Cas9 and *CDKN1B/EGFP* donor were electroporated into cells using Cell Line Nucleofector® Kit R (25 RCT) (Lonza, VCA-1001) via the Nucleofector II (Lonza) with 4 pulses, program G-016. The knock-in single cells were selected by addition of puromycin (2 μg/ml) for 24 h and followed by fluorescence-activated cell sorting (FACS). The inserted elements within genomic DNA of the *CDKN1B/EGFP* knock-in single cell clones were validated by PCR and Sanger sequencing (Table S1 and Figure S2) and the EGFP fluorescence were verified by flow cytometry and immunofluorescence. The sgRNA expression vector containing Cas9 and *MKI67/mCherry* donor were then electroporated into the verified *CDKN1B/EGFP* knock-in cell clone (s) and the dual genome-editing cell clones were generated using the same approach above.

### Reagents

The information of reagents used in this study is provided in Table S2.

### Fluorescence imaging

The dually edited cells were seeded onto coverslip in 24-well plates overnight. Cells were fixed in 1% paraformaldehyde for 10 min. After washing with PBS, the nucleus was stained with 2-(4-Amidinophenyl)-6-indolecarbamidine dihydrochloride (DAPI) for 10 min. Epifluorescence images were captured using Axio Imager equipped with a 40× objective (Carl Zeiss).

### Western blotting

Western blotting was carried out as described previously 8. Information of antibodies used in this study is provided in Table S3.

### Cell sorting

The dually edited cells in the presence or absence of indicated treatments were suspended in cell sorting buffer (5 mM EDTA, 25 mM HEPES and 1% FCS in PBS) and subjected to FACSAria III (BD Biosciences). The sorted quiescent and cycling compartments were then confirmed using Hoechst 33342/Pyronin Y staining and subjected to following studies.

### Bromodeoxyuridine (BrdU) incorporation

EGFP-p27^high^mCherry-Ki67^low^ quiescent (Q) and cycling (C) cells seeded in 96-well plates (5 × 10^3^ cells per well) were subjected to DNA synthesis assays using the BrdU Assay kit (Cell Signalling) as described previously 22. Absorbance was read at 450 nm using a Synergy™ 2 multidetection microplate reader (BioTek, VT).

### Cell-cycle analysis

Hoechst 33342/Pyronin Y double staining were performed as described previously 8.

### Three-dimensional (3-D) culture

The 3D culture was performed using the hanging drop technique 23. Briefly, three hundred of the indicated cells were hung on the lid of a 100 mm dish for 7 days until the 3D spheroids were formed. The spheroids were then treated by serum free medium or indicated inhibitors for further 24 h before photograph taken.

### RNA sequencing

Total RNA from the isolated quiescent and cycling cells was isolated using the ISOLATE II RNA Mini Kit (Bioline, BIO-52073) according to the manufacturer's instructions. The extracted RNA samples were dried to powder in RNA stable tubes (Biomatrica, San Diego, CA) using Rotational Vacuum Concentrator (MARTIN CHRIST, RVC 2-25). The biological duplicated RNA samples were delivered and subjected to transcriptome sequencing, and then analyzed by the Genewiz (Suzhou, China). The data were deposited in the Gene Expression Omnibus (GEO) under accession code GSE174520.

### Quantitative PCR (qPCR)

Total cellular RNA was reverse transcribed to cDNA using the qScript cDNA Supermix (Quantabio, 95048), and then subjected to qPCR using SensiFAST SYBR Hi-ROX Kit (Bioline). The 2^-ΔΔCT^ method was used to calculate the relative gene expression levels in comparison with housekeeping controls. Primers used are detailed in Table S4.

### Single-cell RNA-seq (scRNA-seq)

The dually edited Mel-RM (Mel-RM.DE) cells were serum starved for 96 h and the EGFP-p27^high^mCherry-Ki67^low^ quiescent cells were sorted, washed twice and resuspended in cold PBS (calcium and magnesium free) with 0.04% FBS. Cell number and viability were determined using hemocytometer and Trypan Blue staining and 1 × 10^5^ cells were subjected to 10× Genomics sequencing according to the manufacturer's protocol by Shanghai OE Biotech co., LTD (Shanghai, China) 24. Briefly, viable EGFP-p27^high^mCherry-Ki67^low^ cells isolated from dually edited Mel-RM cells after serum-starvation were analyzed using the 10× Genomics Chromium Droplet platform with unique transcript counting through barcoding with unique molecular identifiers (UMIs). Cell Ranger 3.1.0 and Seurat 3.1.1 were used to analyze the sequencing results. After quality filtering to remove cells expressing high mitochondrial gene signatures and excluding doublets, 7544 cells were retained for further analysis. Upon gene expression normalization for read depth and mitochondrial read counts, cells were subjected to principal component analysis on genes differentially expressed. A total of 7 unsupervised cell clusters were obtained using graph-based clustering on the informative principal components and were visualized using Loupe Browser. Loupe Browser was also used to re-cluster the cells arbitrarily into two clusters according to whether they expression a panel of cell cycle progression genes (*CCNA2, CCNB1, CCNB2, CDC20, CDCA8* and* PLK1*).

### Seahorse extracellular flux (Seahorse XF) assays

Assays were performed using the Seahorse XFe96 analyzer (Seahorse Bioscience, Agilent) according to the manufacturer's instructions. The oxygen consumption rate (OCR) was measured using a Seahorse XF Cell Mito Stress Test Kit (Seahorse Bioscience, Agilent). Briefly, the sorted quiescent and cycling cells were seeded on Seahorse XF Cell Culture Microplate with assay medium (Seahorse XF Base Medium with 1 mM pyruvate, 2 mM glutamine, and 10 mM glucose. pH = 7.4) for 1 h at 37 °C without CO_2_. The OCR was measured under basal conditions and after the addition of oligomycin (1 μM), FCCP (1 μM), and rotenone (0.5 μM)/antimycin (0.5 μM). The extracellular acidification rate (ECAR) was measured using a Seahorse XF Glycolysis Stress Test Kit (Seahorse Bioscience, Agilent). Briefly, the isolated quiescent and cycling cells were seeded on Seahorse XF Cell Culture Microplate with assay medium (Seahorse XF Base Medium with 2 mM glutamine. pH = 7.4) for 1 h at 37 °C without CO_2_. The ECAR was measured under basal conditions and after the addition of glucose (10 mM), oligomycin (1 μM), and 2-DG (50 mM).

### ROS measurements

ROS levels were measured using CellROX™ Deep Red Reagent (Thermo Fisher, C10422) as per the manufacturer's protocol. Briefly, the isolated quiescent and cycling cells were incubated with DMEM containing 500 nM CellROX® Deep Red reagents for 30 min. The ROS levels were then detected by LSRFORTESSA X-20 (BD Biosciences).

### Lactate, IDH activity, α-KG, NADH/NAD^+^ ratio measurements

Cellular lactate levels, α-KG levels, IDH1/IDH2 and IDH3 activities, and NADH/NAD^+^ ratios were measured using the Lactate Assay Kit (Sigma, MAK064), α-Ketoglutarate Assay Kit (Sigma, MAK054), Isocitrate Dehydrogenase Activity Assay Kit (Sigma, MAK062), and NAD/NADH Quantification Kit (Sigma, MAK037), respectively, according to the manufacturer's instructions. The measurements were performed and recorded by a Synergy 2 multidetection microplate reader (BioTek, USA).

### Propidium iodide (PI) uptake assays

For PI uptake assays, cells with indicated treatment were washed twice by PBS and subjected to PI staining for 15 min. The dead cells were analysed using a flow cytometer (FACSCanto II; BD Biosciences) 25.

### Cell viability assays

Cell viability was measured using Cell Counting Kit 8 (WST-8/CCK8, Abcam, ab228554) according to the manufacturer's instructions. Briefly, cells were seeded at 5 × 10^3^/well in 96-well plates overnight before experimental treatments. WST-8 solution (10 µl) was added and incubated at 37 °C for 2 h. The absorbance at 450 nm was then recorded by Synergy 2 multidetection microplate reader (BioTek, USA).

### Inducible knockdown

EZ-Tet-pLKO-Puro vector (Addgene, #85966) were digested by NheI-HF (NEB, R3131) and EcoRI-HF (NEB, R3101) and purified using ISOLATE II PCR and Gel Kit (Bioline, BIO-52060). The linearized vectors were ligated with the annealed double-strand shRNA (Table S5) and then confirmed by Sanger sequencing. The EZ-Tet-pLKO-Puro-shRNA plasmids were co-transfected with pMDLg.pRRE, pRSU.pREV, and pMD2.g to HEK293T cells and the packaged lentivirus were harvested 48 h later. The dually edited cells were then transduced with lentiviral particles carrying inducible c-Myc or IDH3A shRNAs and the inducible knockdown cell sublines were selected using 2 μg/ml puromycin.

### Chromatin immunoprecipitation (ChIP) assays

ChIP assays were performed as previously described 8. Briefly, the isolated quiescent and cycling cells (5 × 10^6^ of each) were cross-linked with a final concentration of 1% formaldehyde in PBS for 10 min at room temperature (RT). The cross-linking was then quenched by the addition of glycine to a final concentration of 125 mM and incubation for 5 min at RT. Cells were rinsed twice with cold PBS and harvested in Lysis Buffer with protease inhibitors and sonicated to shear the chromatin to yield DNA fragment sizes at 200 to 500 bp. Samples were then centrifuged at 12,000 g for 10 min at 4 °C. A portion of the precleared samples were used as input DNA. The antibodies were coupled with the Dynabeads® before sonication at 4 °C for 1 h and approximately 10 μl of c-Myc antibody (Table S3) or 1 μl rabbit normal immunoglobulin (IgG) was used for each IP. The cleared samples were incubated for 2 h with the pre-coupled antibody-Dynabeads® complexes. Beads were washed three times by IP buffer 1 and IP buffer 2 of each. Then the formaldehyde crosslinking in the washed sample was reversed. The DNA was purified for further qPCR test. Control IgG and input DNA signal values were used to normalize the values from the Myc ChIP to target genes. The primers for target genes are listed in Table S6.

### Luciferase reporter assays

C-Myc binding region(s) at *IDH3A, IDH3B, IDH3G, CCNA2, CDC20* and *CDC45* gene promoters were analyzed according to ENCODE TFBS ChIP-seq data. The promoters including c-Myc binding region(s) were amplified from Mel-RM genomic DNA using PCR and then constructed to HindIII enzyme linearized pGL3 Basic vector (Promega, #E1751) using NEBuilder® HiFi DNA Assembly Cloning Kit (NEB, E5520). Primers used are listed in Table S7. The dually edited cells were serum staved for 72 h and then co-transfected with individual pGL3-promoter plasmids expressing firefly luciferase and pRL-SV40P vector expressing Renila luciferase. The quiescent and cycling dually edited cells were sorted 24 h after transfection and Firefly and Renilla luciferase activities were detected using a microplate reader (BioTek, USA).

### Statistical analysis

Statistical analysis was carried out using GraphPad Prism 8. Statistical significance was analyzed by two-tailed Student's *t*-test or ANOVA and expressed as a *P* value. *P* < 0.05 was considered to be statistically significance.

## Results

### Dual genome-editing of endogenous p27 and Ki67 allows for visualization and isolation of p27^high^Ki67^low^ quiescent cancer cells

**Figure 1:**
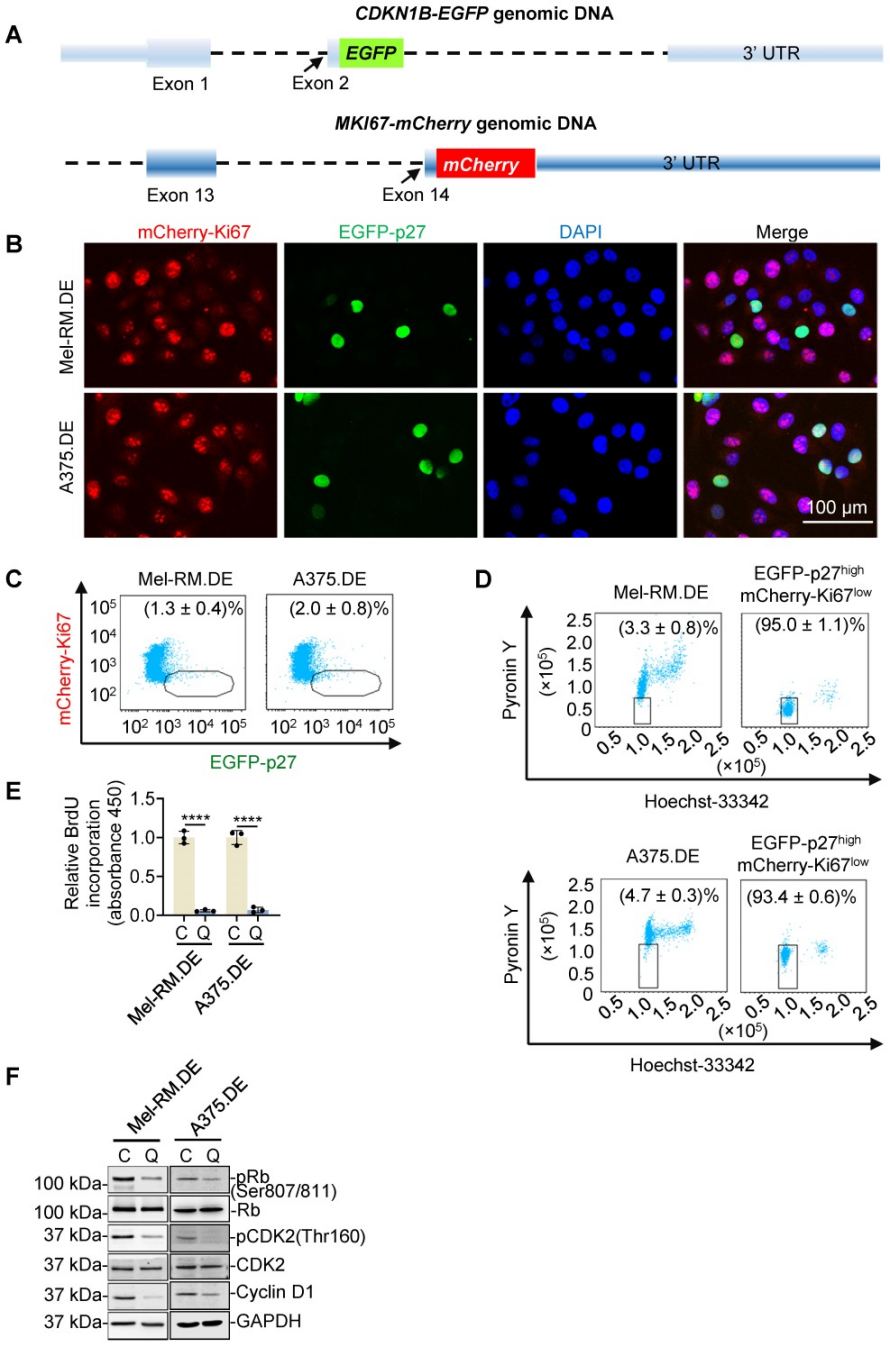
** Dual genome-editing of endogenous p27 and Ki67 to identify EGFP-p27^high^mCherry-Ki67^low^ quiescent cancer cells. (A)** Schematic illustration of fusing *EGFP* and *mCherry* sequences at the C-termini of the *CDKN1B* and *MKI67* genes, respectively, using the CRISPR-Cas9 system. **(B)** Representative microscopic photographs of dually edited Mel-RM (Mel-RM.DE) and A375 (A375.DE) cells with nuclei labelled with DAPI. Scale bar, 100 μm;* n* = 3. **(C)** Representative flowcytometry dot plots showing that a small proportion of Mel-RM.DE and A375.DE cells were EGFP-p27^high^mCherry-Ki67^low^, Values are mean ± SDs; *n* = 3. **(D)** Representative flowcytometry dot plots of EGFP-p27^high^mCherry-Ki67^low^ cells isolated using FACS from Mel-RM.DE and A375.DE cells with DNA and RNA labelled using hoechst-33342 and pyronin Y, respectively. Values are mean ± SDs; *n* = 3. **(E)** DNA synthesis activity was not detected in EGFP-p27^high^mCherry-Ki67^low^ [quiescent cells (Q)] cells, but was readily detectable in the other [cycling (C)] cells isolated from Mel-RM.DE and A375.DE cells as shown in BrdU incorporation assays. The relative BrdU incorporation in cycling cells were arbitrarily designated as 1. Values are mean ± SDs; *n* = 3 (****P < 0.0001, two-tailed Student's *t*-test). **(F)** Whole cell lysates from EGFP-p27^high^mCherry-Ki67^low^ quiescent (Q) and the cycling cells isolated from Mel-RM.DE and A375.DE cells were analyzed using Western blotting. *n* = 3.

As quiescent cells characteristically express high levels of p27 and low levels of Ki67 12, 13, we reasoned that cells with endogenous p27 and Ki67 labelled with differentiable fluorescent probes would allow for isolation of viable quiescent cells. By use of CRISPR-Cas9 genome editing, we introduced sequences encoding EGFP at the endogenous locus of *CDKN1B* and sequences encoding mCherry at the endogenous locus of *MKI67*, into Mel-RM and A375 melanoma cells (Figure 1A and Figure S1). Appropriate targeting of *CDKN1B* and* MKI67* was confirmed using PCR and Sanger sequencing (Figure S2). EGFP-labelled p27 (EGFP-p27) and mCherry-labelled Ki67 (mCherry-Ki67) displayed the expected nuclear localization and were expressed at levels similar to those of unlabelled p27 and Ki67 in unedited parental cells, respectively (Figure 1B and Figure S3) 15, 26. Importantly, the proliferation rate and cell cycle distribution of the edited cells were not altered (Figure S4), suggesting that this labelling of endogenous p27 and Ki67 did not affect their functions. Under steady-state conditions, a small proportion of the cells appeared EGFP-p27^high^mCherry-Ki67^low^, suggestive of spontaneous quiescence (Figure 1B, C). Dual nucleic acid staining (DNA with Hoechst-33342, and RNA, Pyronin Y) showed that isolated EGFP-p27^high^mCherry-Ki67^low^ cells were diploid and contained low RNA content (Figure 1D), whereas BrdU incorporation assays revealed that DNA synthesis activity was not detectable in isolated EGFP-p27^high^mCherry-Ki67^low^ cells (Figure 1E), corroborating their quiescent state 27. In support, EGFP-p27^high^mCherry-Ki67^low^ cells exhibited minimally phosphorylated levels of the cell proliferation markers retinoblastoma protein (pRB) and cyclin-dependent kinase 2 (pCDK2) along with cyclin D1 (Figure 1F).

**Figure 2:**
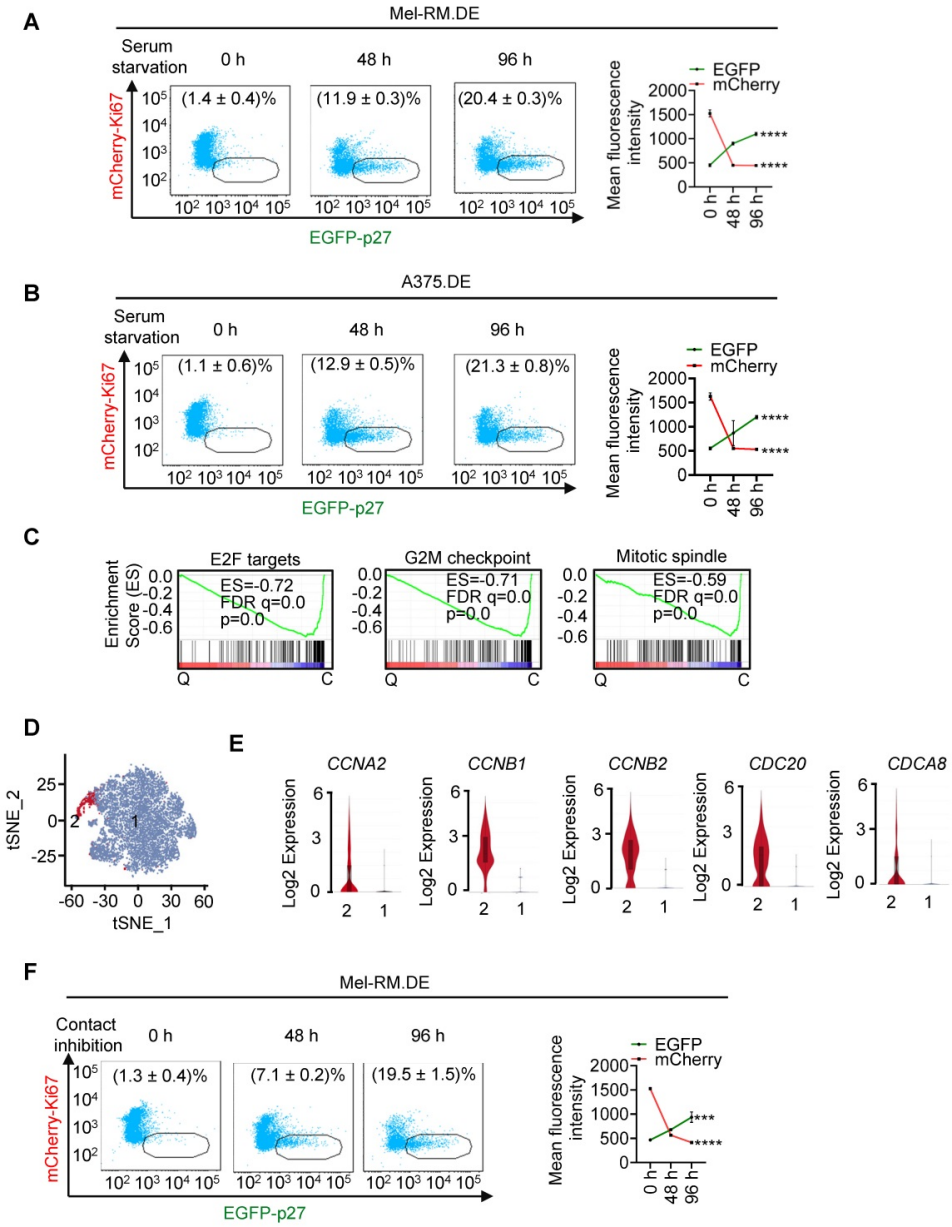
** Isolation and characterization of EGFP-p27^high^mCherry-Ki67^low^ cells enriched by serum starvation or contact inhibition. (A, B)** Dually edited Mel-RM (Mel-RM.DE; A) and A375 (A375.DE; B) cells undergoing serum starvation for indicated periods were subjected to flowcytometry. The EGFP-p27^high^mCherry-Ki67^low^ quiescent cell population was gated and the percentage of these cells calculated (left panel). Mean fluorescence intensities of EGFP and mCherry were also quantitated and shown. Values are mean ± SDs; *n* = 3 (****P < 0.0001, One-way ANOVA). **(C)** GSEA plots of bcRNA-seq data from Mel-RM.DE cells undergoing serum starvation showing that the E2F, G2M progression and mitotic spindle assembly pathways were negatively enriched in EGFP-p27^high^mCherry-Ki67^low^ quiescent (Q) compared with cycling (C) cells. FDR, false-discovery rate; ES, enrichment score. *n* = 2 biological repeats. **(D)** t-Distributed stochastic neighbor embedding (t-SNE) visualization of transcriptomes of 7554 single EGFP-p27^high^mCherry-Ki67^low^ quiescent cells isolated from Mel-RM.DE cells undergoing serum starvation. The cells were arbitrarily clustered into cluster 1 and 2 that did not and did express cell cycle progression genes including *CCNA2, CCNB1, CCNB2, CDC20* and* CDCA8,* respectively. **(E)** Violin plots showing the smoothened expression distribution of *CCNA2, CCNB1, CCNB2, CDC20* and* CDCA8*, stratified per the two clusters showed in (D). **(F)** Dually edited Mel-RM cells undergoing contact inhibition for indicated periods were subjected to flowcytometry. The EGFP-p27^high^mCherry-Ki67^low^ quiescent cell population was gated and the percentage of these cells calculated. Values are mean ± SDs; *n* = 3. Mean fluorescence intensities of EGFP and mCherry were also quantitated and shown. Values are mean ± SDs; *n* = 3 (***P < 0.001; ****P < 0.0001, One-way ANOVA).

To further test the suitability of EGFP-p27 and mCherry-Ki67 as markers for identifying quiescent cells, we subjected dually edited Mel-RM and A375 cells to serum starvation for up to 96 h. There were progressively increases in the EGFP-p27^high^mCherry-Ki67^low^ population, whereas the fraction of the other cells (cycling cells) was concurrently reduced (Figure 2A, B), mirroring induction of cellular quiescence. Similar to spontaneous EGFP-p27^high^mCherry-Ki67^low^ cells (Figure 1D-F), EGFP-p27^high^mCherry-Ki67^low^ cells enriched by serum starvation were diploid, displayed low RNA content, exhibited no DNA synthesis activity, and expressed low levels of pRB, cyclin D1 and pCDK2 compared with cycling cells (Figure S5). Moreover, gene set enrichment analysis (GSEA) of transcriptomes obtained through bulk cell RNA-sequencing (bcRNA-seq) of dually edited Mel-RM cells showed that the E2F, G2M progression and mitotic spindle assembly pathways were markedly weakened in EGFP-p27^high^mCherry-Ki67^low^ cells (Figure 2C).

We also carried out single cell RNA-seq (scRNA-seq) analysis of EGFP-p27^high^mCherry-Ki67^low^ cells isolated from dually edited Mel-RM cells after serum-starvation. After quality filtering to remove cells expressing high mitochondrial gene signatures and cell doublets, 7544 cells were retained for further analysis 28. Although these cells could be grouped into 7 unsupervised clusters after principal component analysis, the t-distributed stochastic neighbour embedding (t-SNE) plot showed a rather poor separation of the clusters (Figure S6), implicating that they represent various cell states on an ordered scale rather than separate entities 29. Indeed, the vast majority of the cells expressed no or negligible levels of many key genes involved in cell cycle progression, whereas a small subset of the cells (371 of 7544 cells; ~5%) expressed readily detectable levels of cell cycle progression genes, such as *CCNA2, CCNB1, CCNB2, CDC20,* and *CDCA8*, conceivably representing cells at different depths of quiescence or reflecting imperfections inherent in the isolation process 2, 30-32. We accordingly re-clustered the cells into two clusters in an arbitrary manner (Figure 2D, E and Figure S7).

Noticeably, bcRNA-seq showed upregulation of the *TP53* gene along with many p53 transcriptional target genes in EGFP-p27^high^mCherry-Ki67^low^ quiescent cells (Figure S8), consistent with the involvement of p53 in regulating cellular quiescence 8, 10. In accord, these genes were readily detectable in most EGFP-p27^high^mCherry-Ki67^low^ quiescent cells as shown in scRNA-seq analysis (Figure S8C). We validated the differential expression of subsets of genes selected to represent the E2F, G2M progression, mitotic spindle assembly and p53 pathways in EGFP-p27^high^mCherry-Ki67^low^ quiescent cells and cycling cells (Figure S9). Furthermore, the proportion of EGFP-p27^high^mCherry-Ki67^low^ cells were progressively increased in dually edited Mel-RM cells subjected to contact inhibition, another commonly used experimental approach for induction of cellular quiescence (Figure 2F) 33. Taken together, these results indicate that endogenous p27 and Ki67 labelled with differentiable fluorescent proteins are reliable markers allowing for unbiased, sensitive visualization and isolation of p27^high^Ki67^low^ quiescent cells.

### Metabolic switching towards mitochondrial OXPHOS in quiescent cells

**Figure 3:**
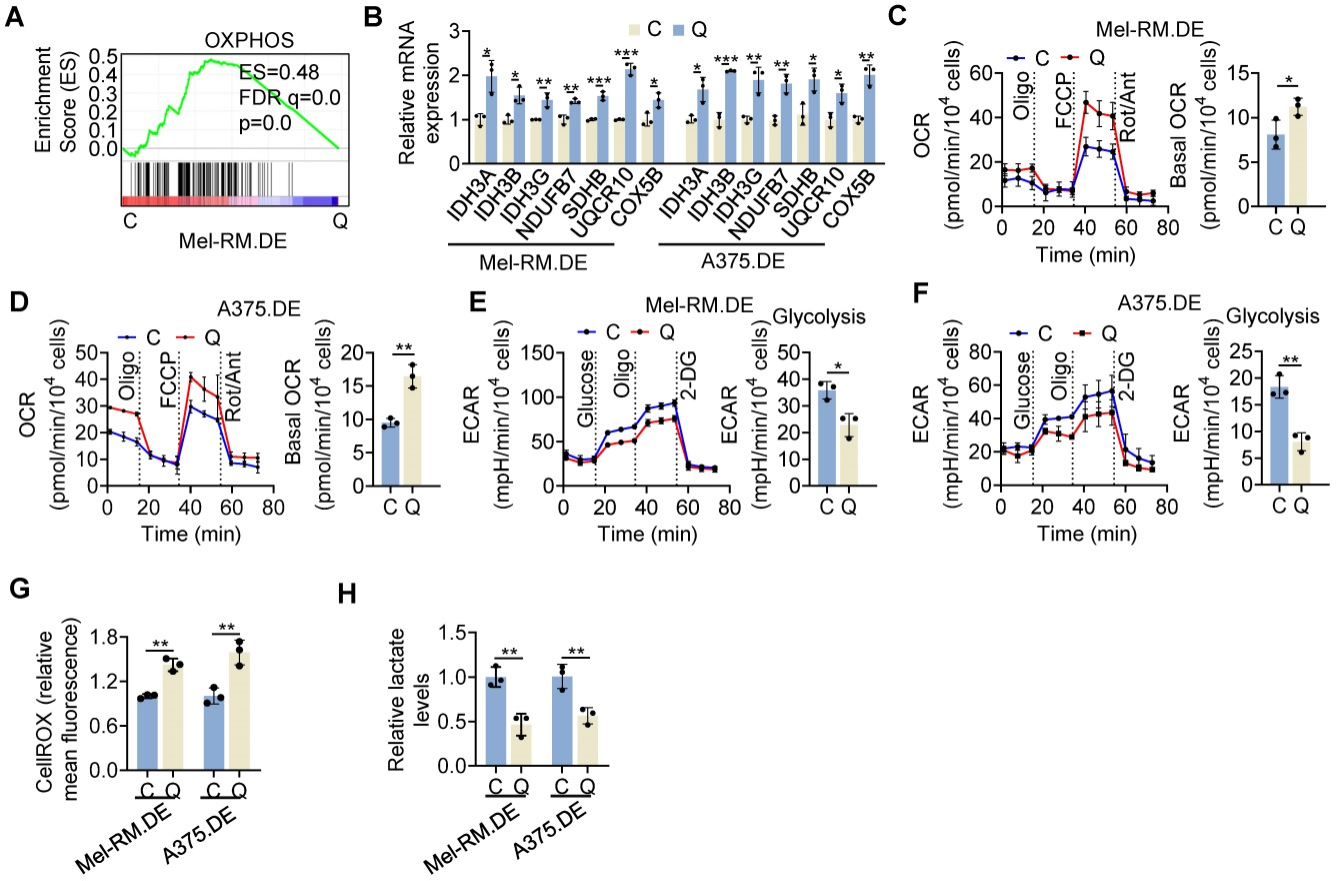
** High OXPHOS activity in quiescent melanoma cells. (A)** A GSEA plot of RNA-seq data showing that the OXPHOS pathway was enriched in EGFP-p27^high^mCherry-Ki67^low^ (Q) compared with cycling (C) cells isolated from dually edited Mel-RM cells (Mel-RM.DE) undergoing serum starvation. ES, enrichment score; FDR, false-discovery rate. *n* = 2 biological repeats. **(B)** Total RNA from EGFP-p27^high^mCherry-Ki67^low^ quiescent (Q) and cycling (C) cells isolated from Mel-RM.DE and A375.DE cells undergoing serum starvation were subjected to qPCR analysis. Values are mean ± SDs; *n* = 3 (*P < 0.05; **P < 0.01; ***P < 0.001, two-tailed Student's *t*-test). **(C, D)** EGFP-p27^high^mCherry-Ki67^low^ quiescent (Q) and cycling (C) cells isolated from Mel-RM.DE (C) and A375.DE (D) cells undergoing serum starvation were subjected to Seahorse XF analysis of the OCR. Values are mean ± SDs; *n* = 3 (*P < 0.05; **P < 0.01, two-tailed Student's *t*-test ). **(E, F)** EGFP-p27^high^mCherry-Ki67^low^ quiescent (Q) and cycling (C) cells isolated from Mel-RM.DE (E) and A375.DE (F) cells undergoing serum starvation were subjected to Seahorse XF analysis of the extracellular acidification rate (ECAR). Values are mean ± SDs; *n* = 3 (*P < 0.05; **P < 0.01, two-tailed Student's *t*-test). **(G)** EGFP-p27^high^mCherry-Ki67^low^ quiescent (Q) and cycling (C) cells isolated from Mel-RM.DE and A375.DE cells undergoing serum starvation were subjected to CellROX analysis. Values are mean ± SDs; *n* = 3 (**P < 0.01, two-tailed Student's *t*-test). **(H)** EGFP-p27^high^mCherry-Ki67^low^ quiescent (Q) and E cycling (C) cells isolated from Mel-RM.DE and A375.DE cells undergoing serum starvation were subjected to colorimetric analysis of intracellular lactate levels. Values are mean ± SDs; *n* = 3 (**P < 0.01, two-tailed Student's *t*-test).

Strikingly, GSEA of bcRNA-seq data revealed that the OXPHOS was among the most enriched pathways in EGFP-p27^high^mCherry-Ki67^low^ quiescent cells isolated from dually edited Mel-RM cells after serum starvation (Figure 3A). The upregulated OXPHOS genes in quiescent cells encompassed those involved in mitochondrial respiratory complexes and the tricarboxylic acid (TCA) cycle (Figure S10). The increases in representative OXPHOS genes (*IDH3A*, *IDH3B*, *IDH3G, NDUFB7, SDHB, UQCR10* and* COX5B*) in quiescent relative to cycling Mel-RM and A375 cells were confirmed using qPCR (Figure 3B). Collectively, these results demonstrate the global upregulation of OXPHOS genes in quiescent cells.

To functionally verify that OXPHOS activity was enhanced in quiescent cells, we performed OCR and ECAR assays, used as proxy measures of mitochondrial respiration and glycolytic flux, respectively 34. As anticipated, EGFP-p27^high^mCherry-Ki67^low^ quiescent Mel-RM and A375 cells displayed increased OCR compared with cycling cells (Figure 3C, D). In contrast, the ECAR was reduced in quiescent cells (Figure 3E, F). Moreover, mitochondrial oxidative stress measured using the CellROX fluorescence assay was increased, whereas the cellular lactate levels were decreased in quiescent Mel-RM and A375 cells (Figure 3G, H). Therefore, metabolic switching towards OXPHOS is a biological trait of cells in quiescence.

### Mitochondrial OXPHOS is critical for survival of quiescent cells

**Figure 4:**
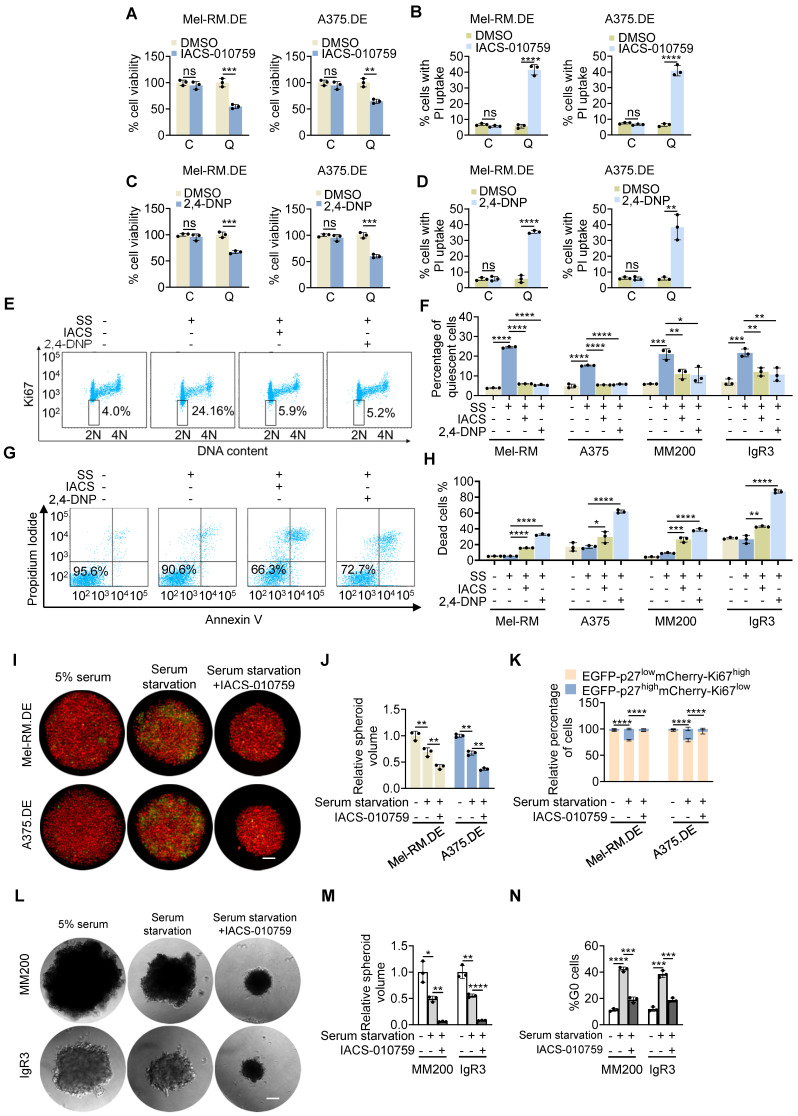
** Quiescent melanoma cells are more reliant on OXPHOS. (A-D)** EGFP-p27^high^mCherry-Ki67^low^ quiescent (Q) and cycling (C) cells isolated from dually edited Mel-RM (Mel-RM.DE) and A375 (A375.DE) cells undergoing serum starvation were treated with IACS-010759 (IACS; 250 nM) (A and B) or 2,4-DNP (250 μM) (C and D) for 24 h before cell viability was measured using the CCK8 assay (A and C), and cell death, the PI uptake assay (B and D). Values are mean ± SDs; *n* = 3 (**P < 0.01; ***P < 0.001; ****P < 0.0001, two-tailed Student's *t*-test ). (**E**) ME4405 cells with or without serum starvation for 72 h treated with IACS-010759 (250 nM) or 2,4-DNP (250 μM) for a further 16 h were subjected to staining with an anti-Ki67 antibody and propidium iodide (PI). Data shown are representative flowcytometry dot plots of three independent experiments. The numbers represent percentages of quiescent cells (diploid cells with no or low levels of Ki67). SS: Serum Starvation. (**F**) Mel-RM, A375, MM200, IgR3 cells with or without serum starvation for 72 h treated with IACS-010759 (250 nM) or 2,4-DNP (250 μM) for a further 16 h were subjected to staining with an anti-Ki67 antibody and propidium iodide (PI). Quiescent cells were quantitated using flowcytometry as exemplified in (E). Values are mean ± SDs; *n* = 3 (*P < 0.05; **P < 0.01; ***P < 0.001; ****P < 0.0001; two-tailed Student's *t*-test). SS: Serum Starvation. (**G**) ME4405 cells with or without serum starvation for 72 h treated with IACS-010759 (250 nM) or 2,4-DNP (250 μM) for a further 48 h were subjected to quantitation of cell death with PI/Annexin V staining using flowcytometry. The numbers represent the relative proportions of viable cells. SS: Serum Starvation. (**H**) Mel-RM, A375, MM200, IgR3 cells with or without serum starvation for 72 h treated with IACS-010759 (250 nM) or 2,4-DNP (250 μM) for a further 48 h were subjected to PI/Annexin V staining. Cell death was quantitated using flowcytometry as exemplified in (G). Values are mean ± SDs; *n* = 3 (*P < 0.05; **P < 0.01; ***P < 0.001; ****P < 0.0001; two-tailed Student's *t*-test). SS: Serum Starvation. **(I)** Representative microscopic photographs showing that treatment with IACS reduced the size of tumour spheres and the proportion of EGFP-p27^high^mCherry-Ki67^low^ quiescent cells of Mel-RM.DE and A375.DE cells undergoing serum starvation. Scale bar, 100 µm; n = 3. **(J)** Quantitative comparison of the size of tumour spheres of Mel-RM.DE and A375.DE cells undergoing serum starvation with or without treatment with IACS. Values are mean ± SDs; *n* = 3 (**P < 0.01, two-tailed Student's *t*-test). **(K)** Quantitative comparison of the relative number of EGFP-p27^high^mCherry-Ki67^low^ quiescent and cycling cells in tumour spheres of Mel-RM.DE and A375.DE cells undergoing serum starvation with or without treatment with IACS. Values are mean ± SDs; *n* = 3 (****P < 0.001, two-way ANOVA). **(L)** Representative microscopic photographs showing that treatment with IACS reduced the size of tumour spheres of MM200 and IgR3 cells undergoing serum starvation. Scale bar, 100 µm. n = 3. **(M)** Quantitative comparison of the size of tumour spheres of MM200 and IgR3 cells undergoing serum starvation with or without treatment with IACS. Values are mean ± SDs; *n* = 3 (*P < 0.05; **P < 0.01; ****P < 0.0001, two-tailed Student's* t*-test). **(N)** Quantitative comparison of the quiescent cells defined using Hoechst-33342 and Pyronin Y staining MM200 and IgR3 cells derived from tumour spheres undergoing serum starvation with or without treatment with IACS. Values are mean ± SDs; *n* = 3 (***P < 0.001; ****P < 0.0001, two-tailed Student's* t*-test).

To examine the functional significance of the increased mitochondrial OXPHOS in quiescent cells, we treated EGFP-p27^high^mCherry-Ki67^low^ quiescent and cycling Mel-RM and A375 cells with IACS-010759 (IACS), a small-molecule inhibitor of mitochondrial complex I 35. The results showed that treatment with IACS for 24 h reduced cell viability in quiescent cells, whereas it did not significantly affect the viability of cycling cells (Figure 4A). The reduction in cell viability in quiescent cells was associated with perturbations of plasma membrane integrity as shown in PI uptake assays (Figure 4B), indicative of necrotic cell death 36. Similarly, quiescent Mel-RM and A375 cells were markedly more sensitive to killing caused by treatment with 2,4-dinitrophenol (DNP), an organic compound that acts as a proton ionophore to uncouple OXPHOS (Figure 4C, D) 37.

We confirmed the role of OXPHOS in supporting quiescent survival of melanoma cells by exposing parental Mel-RM and A375 and a panel of other melanoma cell lines (MM200, IgR3, ME4405) to IACS and DNP in culture media with or without prior deprivation of serum. Treatment with IACS and DNP reduced the percentage of quiescent cells resulting from serum starvation as measured by flow cytometric analysis of Ki-67 and DNA content in all the melanoma cell lines (Figure 4E, F) 27. This was associated with increased levels of cell death (Figure 4G, H), consistent with preferential killing of quiescent cells by the inhibitors (Figure 4B and D).

To test the potential of OXPHOS in regulating the survival of quiescent melanoma cells in tumour spheroids, we deprived dually edited Mel-RM and A375 cells grown in 3-D cultures of serum followed by treatment with IACS. Serum starvation caused an increase in the EGFP-p27^high^mCherry-Ki67^low^ population and a moderate reduction in the size of tumour spheres (Figure 4I-K), indicative of induction of cellular quiescence. Subsequent treatment with IACS resulted in a further decrease in the size of tumour spheres, which was associated with a reduction in the EGFP-p27^high^mCherry-Ki67^low^ population, whereas the percentage of cycling cells was increased (Figure 4I-K), demonstrating preferential killing of quiescent cells in tumour spheres by OXPHOS inhibition. Consistently, exposure of MM200 and IgR3 cells grown in 3-D cultures to IACS following serum starvation decreased the size of tumour spheres, reduced the proportion of quiescent cells and increased the percentage of cycling cells as shown using dual nucleic acid staining (Figure 4L-N).

### c-Myc drives OXPHOS in quiescent cells

**Figure 5:**
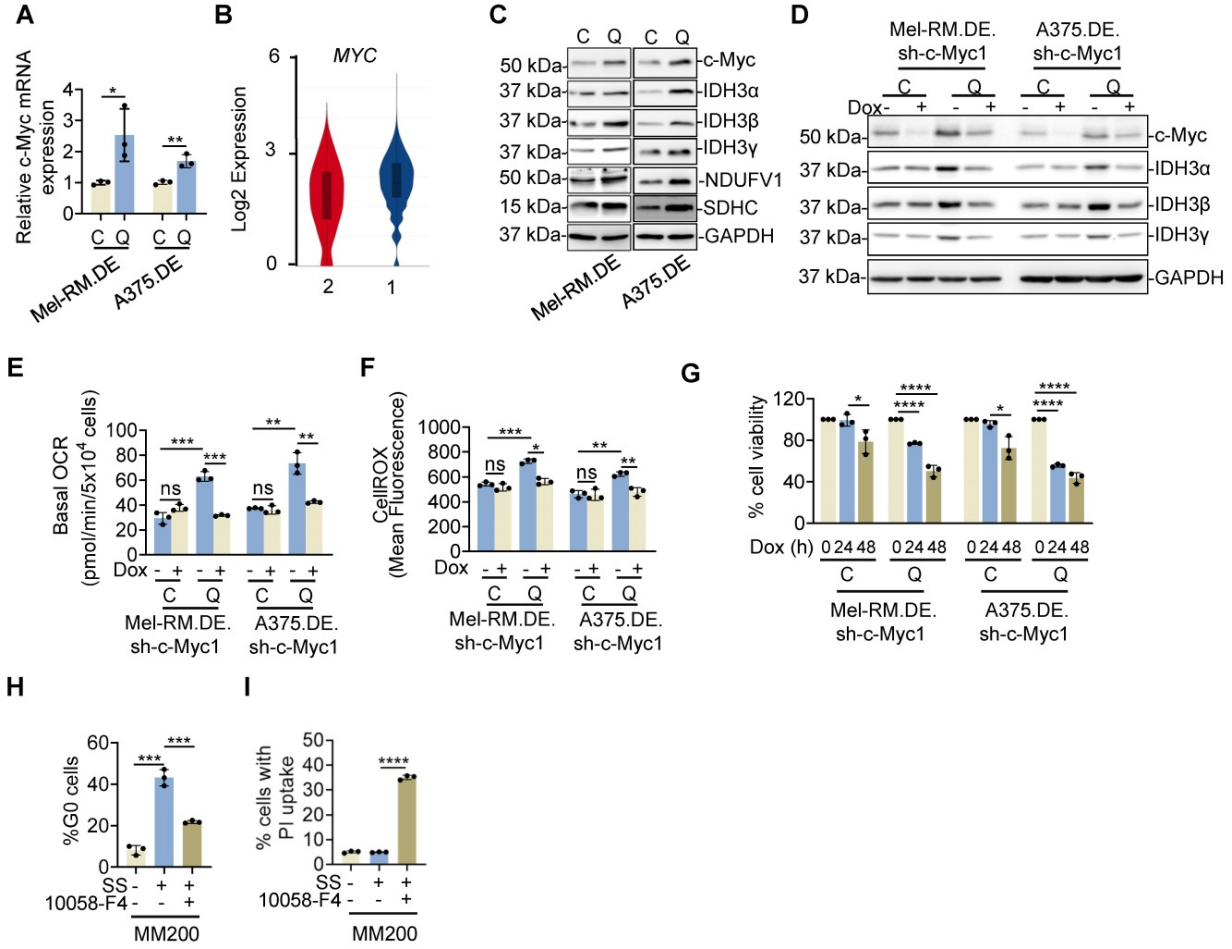
** c-Myc drives OXPHOS in quiescent melanoma cells. (A)** Total RNA from EGFP-p27^high^mCherry-Ki67^low^ quiescent (Q) and cycling (C) cells isolated from dually edited Mel-RM (Mel-RM.DE) and A375 (A375.DE) cells undergoing serum starvation were subjected to qPCR analysis of c-*MYC* mRNA expression. Values are mean ± SDs; *n* = 3 (*P < 0.05; **P < 0.01, two-tailed Student's *t*-test ). **(B)** Violin plots showing the smoothened expression distribution of *MYC* stratified per the two clusters showed in Figure 2D. **(C)** Whole cell lysates from EGFP-p27^high^mCherry-Ki67^low^ quiescent (Q) and cycling (C) cells isolated from Mel-RM.DE and A375.DE cells undergoing serum starvation were analyzed using Western blotting. *n* = 3. **(D)** Whole cell lysates from EGFP-p27^high^mCherry-Ki67^low^ quiescent (Q) and cycling (C) cells isolated from Mel-RM.DE and A375.DE cells carrying an inducible c-Myc shRNA system with or without treatment with Doxycycline (Dox) for 24 h undergoing serum starvation were subjected to Western blotting. *n* = 3. **(E)** EGFP-p27^high^mCherry-Ki67^low^ quiescent (Q) and cycling (C) cells isolated from Mel-RM.DE and A375.DE cells carrying an inducible c-Myc shRNA system with or without treatment with Dox undergoing serum starvation were subjected to Seahorse XF analysis of the oxygen consumption rate (OCR). Values are mean ± SDs; n = 3 (**P < 0.01; ***P < 0.001, two-tailed Student's *t*-test). **(F)** EGFP-p27^high^mCherry-Ki67^low^ quiescent (Q) and cycling (C) cells isolated from Mel-RM.DE and A375.DE cells carrying an inducible c-Myc shRNA system with or without treatment with Dox undergoing serum starvation were subjected to CellROX analysis. Values are mean ± SDs; *n* = 3 (*P < 0.05; **P < 0.01; ***P < 0.001, two-tailed Student's *t*-test). **(G)** EGFP-p27^high^mCherry-Ki67^low^ quiescent (Q) and cycling (C) cells isolated from Mel-RM.DE and A375.DE cells carrying an inducible c-Myc shRNA system undergoing serum starvation were treated with Dox for the indicated periods were subjected to measurement of cell viability using CCK8 assays. The relative viability of quiescent and cycling cells of each cell line without treatment with Dox was arbitrarily designated as 100%, respectively. Values are mean ± SDs; *n* = 3 (*P < 0.05; ****P < 0.001, two-tailed Student's *t*-test). **(H)** MM200 cells with or without serum starvation for 72 h were treated with 10058-F4 (50 μM) for an additional 16 h. Quiescent cell proportions were quantitated with Hoechst-33342 and Pyronin Y staining using flowcytometry. Values are mean ± SDs; *n* = 3 (***P < 0.001, two-tailed Student's *t*-test). **(I)** MM200 cells with or without serum starvation for 72 h were treated with 10058-F4 (50 μM) for an additional 48 h. Cell death was quantitated using PI uptake assays. Values are mean ± SDs; *n* = 3 (****P < 0.0001, two-tailed Student's *t*-test).

Having demonstrated the importance of OXPHOS in survival of quiescent cancer cells, we focused on investigation of the mechanism responsible for the increased OXPHOS activity. Intriguingly, *MYC* was among the genes that were expressed at higher levels in quiescent compared with cycling Mel-RM cells as shown by bcRNA-seq (Figure 5A and Figure S11A). *MYC* encodes the proto-oncoprotein c-Myc that plays an important role in driving malignant cell cycle progression 21. Indeed, scRNA-seq analysis revealed that *MYC* was readily detected in EGFP-p27^high^mCherry-Ki67^low^ quiescent Mel-RM cells (Figure 5B). In particular, there was a tendency that cells in cluster 1 that expressed no or negligible levels of cell cycle progression genes expressed higher levels of MYC relative to those in cluster 2 that displayed readily detectable levels of genes involved in cell cycle progression (Figure 2D, E and 5B). Of note, although the c-Myc pathway as a whole was not significantly enriched in quiescent compared with cycling cells (Table S8), the OXPHOS genes responsive to c-Myc were globally increased, which well-overlapped with the increased mitochondrial respiration chain and TCA cycle genes in quiescent cells (Figure S11B). We confirmed the relatively high expression of the c-Myc protein and a number of OXPHOS enzymes (IDH3A, IDH3B, IDH3G, NDUFV1 and SDHC) encoded by c-Myc pathway genes in quiescent Mel-RM and A375 cells using immunoblotting (Figure 5C). Taken together, these results indicate that c-Myc is expressed in quiescent melanoma cells and suggest that c-Myc may be involved in driving OXPHOS in quiescent cells.

To verify the role of c-Myc in the increased OXPHOS activity in quiescent cells, we introduced an inducible c-Myc shRNA system responsive to doxycycline (Dox) into dually edited Mel-RM and A375 cells (Figure S12A). Induced knockdown of c-Myc abolished the increase in representative OXPHOS enzymes, including IDH3α, IDH3β and IDH3γ, at both the mRNA and protein levels in isolated EGFP-p27^high^mCherry-Ki67^low^ quiescent cells (Figure 5D and Figure S12B), whereas overexpression of c-Myc caused further increases in the expression of these enzymes (Figure S12C, D). In contrast, neither knockdown nor overexpression of c-Myc significantly impinged on the expression of IDH3α, IDH3β and IDH3γ in cycling cells (Figure 5D and Figure S12B-D).

Moreover, knockdown of c-Myc diminished the increase in the OCR and mitochondrial oxidative stress and caused reduction in the viability of quiescent cells (Figure 5E-G), recapitulating the effects of treatment with OXPHOS inhibitors (Figure 4A and C). Of note, although knockdown of c-Myc for 24 h did not significantly affect the viability of cycling cells, by 48 h, the viability of cycling cells was reduced. This effect was largely caused by inhibition of cell proliferation as we did not detect significant cell death. Consistently, BrdU incorporation assays revealed a reduction in DNA synthesis in cycling cells with c-Myc knockdown (Figure 5G and Figure S12E, F), consistent with the role of c-Myc in promoting cell cycle progression in cycling cells 21, 38. Similarly, treatment with 10058-F4 for 24 h, a small molecule that inhibits c-Myc-mediated transcriptional activation of gene expression 39, reversed the increase in the OCR and mitochondrial oxidative stress and reduced viability in quiescent cells without significant impact on the survival of cycling cells (Figure S12G-I). Nevertheless, exposure to 10058-F4 for 48 h reduced cycling cell proliferation as shown in BrdU incorporation assays (Figure S12J-L). Thus, c-Myc signalling is critical for promoting OXPHOS activity and cell survival in quiescent cells, whereas it drives proliferation in cycling cells 38. Consistently, treatment with 10058-F4 diminished the increase in the quiescent population and enhanced cell death caused by serum starvation in MM200 cells (Figure 5H, I). Together, these results indicate that c-Myc drives OXPHOS and is important for the survival of quiescent melanoma cells.

### c-Myc selectively transactivates OXPHOS genes in quiescent cells

c-Myc plays an important role in transcriptional activation of many genes that promote cell cycle progression, such as *cyclin-dependent kinase* (*CDK*) 1, *CDC20, CDC45* and *CCNA2*, which were however expressed at low levels or not detected in EGFP-p27^high^mCherry-Ki67^low^ quiescent Mel-RM cells as shown in bcRNA-seq and scRNA-seq analyses (Figure 2D and Figure S7 and S13). This was in sharp contrast to the upregulation of c-Myc-responsive OXPHOS genes in quiescent Mel-RM cells (Figure S10). Nevertheless, the differences between the expression of representative c-Myc-regulated cell cycle progression genes (*CDK*1, *CDC20, CDC45*, *CCNA2* and *CCNB2*) and c-Myc-responsive OXPHOS genes (*IDH3A*, *IDH3B*, *IDH3G, NDUFB7, SDHB* and* COX5B*) in quiescent Mel-RM as well as A375 cells were confirmed using qPCR (Figure 3B and Figure S9A). These results suggest that c-Myc differentially regulates its transcriptional target genes in quiescent and cycling cells.

We also tested the expression of c-Myc and its target genes involved in OXPHOS and cell cycle progression in p27^high^Ki67^low^ quiescent cells and cycling cells isolated from dually edited Mel-RM and A375 cells grown in 3-D cultures after serum starvation. Instructively, we found that quiescent cells from tumour spheroids similarly expressed relatively high levels of c-Myc and the c-Myc-responsive OXPHOS genes, *IDH3A, IDH3B, and IDH3G*, whereas the levels of c-Myc-responsive cell cycle progression genes, including *CDC20, CDC45* and *CCNA2* were lower in p27^high^Ki67^low^ quiescent cells compared to cycling cells (Figure S14). These results suggest that quiescent cancer cells in tumour spheroids also express c-Myc which favourably transactivates its OXPHOS target genes.

**Figure 6:**
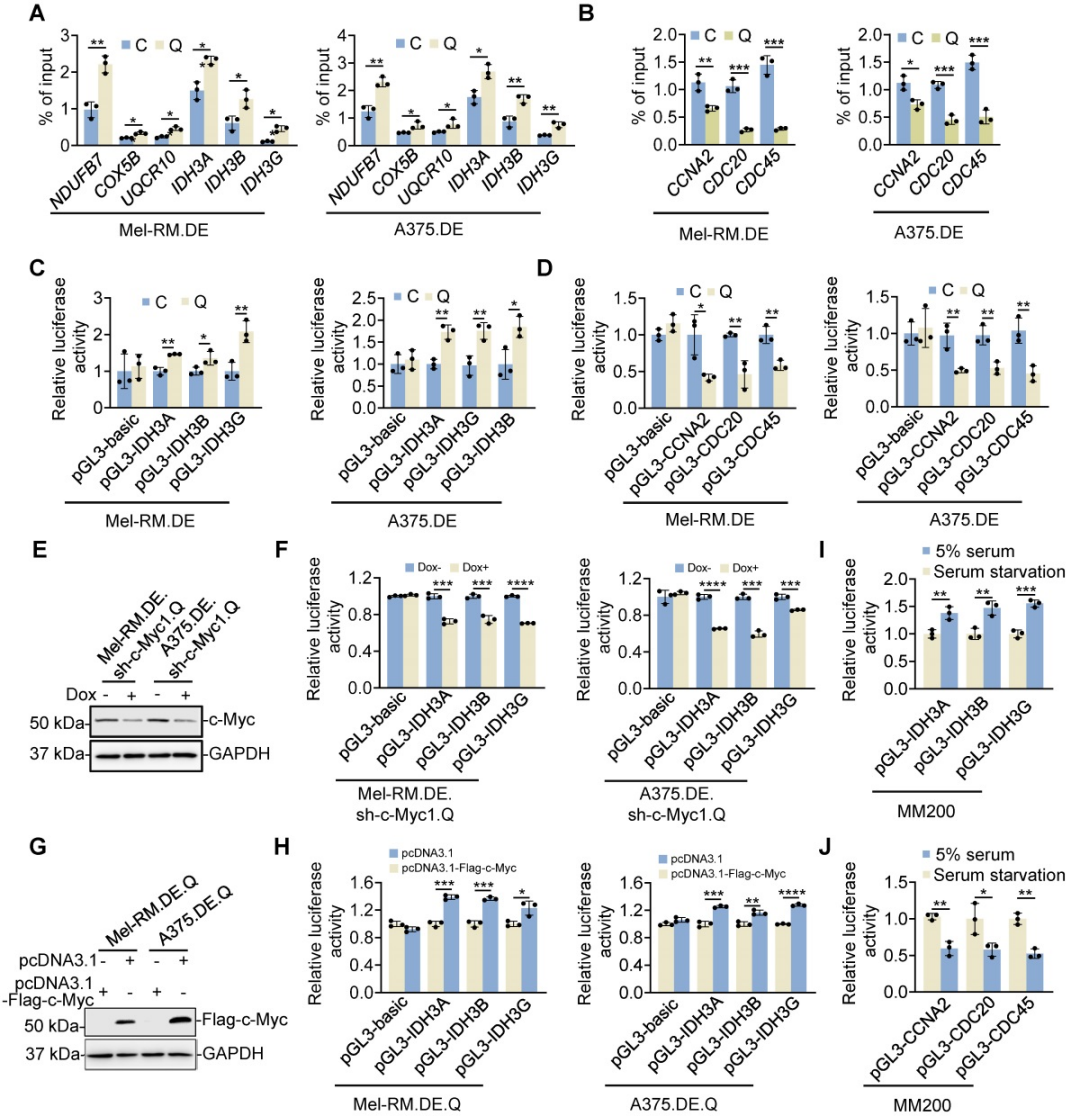
** c-Myc preferentially drives the expression of OXPHOS genes in quiescent cells. (A, B)** EGFP-p27^high^mCherry-Ki67^low^ quiescent (Q) and cycling (C) cells isolated from dually edited Mel-RM (Mel-RM.DE) and A375 (A375.DE) cells undergoing serum starvation were subjected to qPCR-ChIP assays. Values are mean ± SDs; *n* = 3 (*P < 0.05; **P < 0.01, two-tailed Student's *t*-test). **(C, D)** EGFP-p27^high^mCherry-Ki67^low^ quiescent (Q) and cycling (C) cells isolated from Mel-RM.DE and A375.DE cells transfected with the indicated luciferase plasmids undergoing serum starvation were subjected to luciferase reporter assays. Values are mean ± SDs; *n* = 3 (*P < 0.05; **P < 0.01, two-tailed Student's *t*-test). **(E)** Whole cell lysates from EGFP-p27^high^mCherry-Ki67^low^ quiescent (Q) cells isolated from Mel-RM.DE and A375.DE cells carrying an inducible c-Myc shRNA system with or without treatment with Dox undergoing serum starvation were subjected to Western blotting. *n* = 3. **(F)** EGFP-p27^high^mCherry-Ki67^low^ quiescent (Q) cells isolated from Mel-RM.DE and A375.DE cells carrying an inducible c-Myc shRNA system transfected with the indicated luciferase plasmids with or without treatment with Dox were subjected to serum starvation followed by luciferase reporter assays. Values are mean ± SDs; *n* = 3 (***P < 0.001; ****P < 0.00001, two-tailed Student's *t*-test). **(G)** Whole cell lysates from EGFP-p27^high^mCherry-Ki67^low^ quiescent (Q) cells isolated from Mel-RM.DE and A375.DE cells transfected with the indicated plasmids undergoing serum starvation were analyzed using Western blotting. *n* = 3. **(H)** EGFP-p27^high^mCherry-Ki67^low^ quiescent (Q) cells isolated from Mel-RM.DE and A375.DE cells transfected with the indicated plasmids undergoing serum starvation were subjected to luciferase reporter assays. Values are mean ± SDs; *n* = 3 (*P < 0.05; **P < 0.01, ***P < 0.001; ****P < 0.00001, two-tailed Student's *t*-test). **(I, J)** MM200 cells transfected with the indicated luciferase plasmids undergoing serum starvation were subjected to luciferase reporter assays. Values are mean ± SDs; *n* = 3 (*P < 0.05; **P < 0.01; ***P < 0.001, two-tailed Student's *t*-test).

To understand the mechanism through which c-Myc differentially regulates the expression of OXPHOS and cell cycle progression genes in quiescent cells, we tested its physical association with different target gene promoters 40, 41. Chromatin immunoprecipitation (ChIP)-qPCR assays showed that the amounts of c-Myc associated with the promoters of representative OXPHOS genes including *NDUFB7*, *COX5B*, *UQCR10*,* IDH3A*, *IDH3B* and *IDH3G* were increased in quiescent compared with cycling Mel-RM and A375 cells (Figure 6A and Figure S15). In contrast, the amounts of c-Myc binding to the promoters of the representative cell cycle progression genes *CCNA2*, *CDC20,* and* CDC45* were reduced in quiescent cells (Figure 6B). In accordance, the reporter luciferase activities of *IDH3A*, *IDH3B* and *IDH3G* promoters were increased, whereas the reporter luciferase activities of *CCNA2*, *CDC20,* and* CDC45* promoters were decreased, in quiescent compared with cycling Mel-RM and A375 cells (Figure 6C, D). Nevertheless, knockdown of c-Myc diminished the reporter activities of the promoters of *IDH3A, IDH3B* and *IDH3G* in p27^high^Ki67^low^ quiescent cells, whereas the activities of the promoters of *CCNA2, CDC20,* and *CDC45* were reduced in cycling cells when c-Myc was knocked down (Figure 6E, F). In contrast, overexpression of c-Myc increased the reporter activities of the promoters of *IDH3A, IDH3B* and *IDH3G* in p27^high^Ki67^low^ quiescent cells while also driving increased activities of the *CCNA2, CDC20*, and *CDC45* promoters in cycling cells (Figure 6G, H). Therefore, c-Myc selectively drives the expression of OXPHOS genes through preferential occupancy of their promoters in quiescent cells. In support, serum starvation caused increases in the association between c-Myc and *IDH3A*, *IDH3B* and *IDH3G* promoters and decreases in the binding of c-Myc to *CCNA2*, *CDC20,* and* CDC45* promoters in MM200 cells (Figure 6I, J).

### IDH3 upregulation is critical for metabolic switching towards OXPHOS in quiescent cells

Among c-Myc-responsive OXPHOS genes that were upregulated in quiescent compared with cycling Mel-RM cells were *IDH3A*, *IDH3B* and *IDH3G* (Figure 3B and 5C), which encode the three subunits, IDH3α, IDH3β and IDH3γ, respectively, that comprise the NAD (+)-dependent isocitrate dehydrogenase 3 (IDH3) heterotetramer 42. IDH3 catalyzes the rate-limiting step of the TCA cycle through converting isocitrate to α-ketoglutarate (α-KG) 42, 43. As an intermediary product of the TCA, α-KG functions as an electron donor to the oxygen sensors prolyl hydroxylases (PHDs) for prolyl hydroxylation, which is important for hydroxylation and degradation of HIF-1α leading to inhibition of glycolysis 44. Therefore, we examined the potential role of IDH3 in metabolic switching towards OXPHOS in quiescent cells.

**Figure 7:**
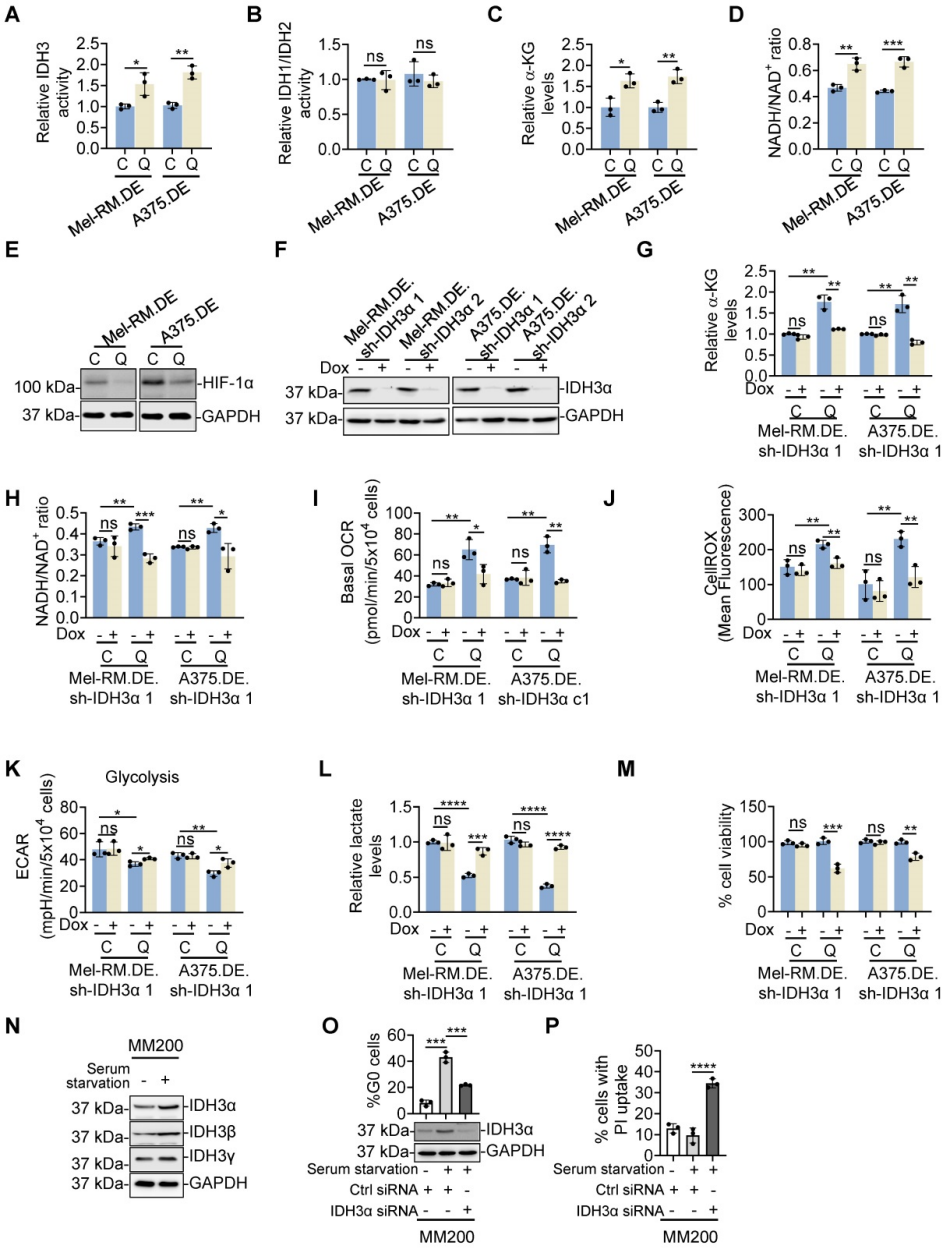
** IDH3 mediates metabolic switching towards OXPHOS in quiescent melanoma cells. (A-D)** EGFP-p27^high^mCherry-Ki67^low^ quiescent (Q) and cycling (C) cells isolated from dually edited Mel-RM (Mel-RM.DE) and A375 (A375.DE) cells undergoing serum starvation were subjected to colorimetric analysis of IDH3 activity (A), IDH1/IDH2 activity (B), α-KG levels (C) and NADH/NAD^+^ ratio (D). Values are mean ± SDs; *n* = 3 (*P < 0.05; **P < 0.01; ***P < 0.01, two-tailed Student's *t*-test). **(E)** Whole cell lysates from EGFP-p27^high^mCherry-Ki67^low^ quiescent (Q) and cycling (C) cells isolated from Mel-RM.DE and A375.DE cells undergoing serum starvation were subjected to Western blotting. *n* = 3. **(F)** Whole cell lysates from Mel-RM.DE and A375.DE cells carrying an inducible IDH3α shRNA system with or without treatment with Dox were subjected to Western blotting. *n* = 3. **(G, H)** EGFP-p27^high^mCherry-Ki67^low^ quiescent (Q) and cycling (C) cells isolated from Mel-RM.DE and A375.DE cells carrying an inducible IDH3α shRNA system with or without treatment with Dox undergoing serum starvation were subjected to colorimetric analysis of α-KG levels (G) and the NADH/NAD^+^ ratio (H). Values are mean ± SDs; *n* = 3 (*P < 0.05; **P < 0.01; ***P < 0.01, two-tailed Student's *t*-test). **(I)** EGFP-p27^high^mCherry-Ki67^low^ quiescent (Q) and cycling (C) cells isolated from Mel-RM.DE and A375.DE cells carrying an inducible IDH3α shRNA system with or without treatment with Dox undergoing serum starvation were subjected to Seahorse XF analysis of the oxygen consumption rate (OCR). Values are mean ± SDs; *n* = 3 (*P < 0.05; **P < 0.01, two-tailed Student's *t*-test). **(J)** EGFP-p27^high^mCherry-Ki67^low^ quiescent (Q) and cycling (C) cells isolated from Mel-RM.DE and A375.DE cells carrying an inducible IDH3α shRNA system with or without treatment with Dox undergoing serum starvation were subjected to CellROX analysis. Values are mean ± SDs; *n* = 3 (**P < 0.01, two-tailed Student's *t*-test). **(K)** EGFP-p27^high^mCherry-Ki67^low^ quiescent (Q) and cycling (C) cells isolated from Mel-RM.DE and A375.DE cells carrying an inducible IDH3α shRNA system with or without treatment with Dox undergoing serum starvation were subjected to Seahorse XF analysis of the extracellular acidification rate (ECAR). Values are mean ± SDs; *n* = 3 (*P < 0.05; **P < 0.01, two-tailed Student's *t*-test). **(L)** EGFP-p27^high^mCherry-Ki67^low^ quiescent (Q) and cycling (C) cells isolated from Mel-RM.DE and A375.DE cells carrying an inducible IDH3α shRNA system with or without treatment with Dox undergoing serum starvation were subjected to colorimetric analysis of intracellular lactate levels. Values are mean ± SDs; *n* = 3 (***P < 0.001; ****P < 0.0001, two-tailed Student's *t*-test). **(M)** EGFP-p27^high^mCherry-Ki67^low^ quiescent (Q) and cycling (C) cells isolated from Mel-RM.DE and A375.DE cells carrying an inducible IDH3α shRNA system were treated with Dox undergoing serum starvation before cell viability was measured using CCK8 assays. Values are mean ± SDs; *n* = 3 (**P < 0.01; ***P < 0.01, two-tailed Student's *t*-test). **(N)** Whole cell lysates from MM200 cells with or without serum starvation for 96 h were subjected to Western blotting. *n* = 3. **(O, P)** MM200 cells transfected with indicated siRNAs were cultured with or without serum starvation. Ninety-six h later, cells were subjected to Western blotting (O, bottom), Hoechst-33342 and Pyronin Y double staining (O, upper), and PI uptake assays (Q). Values are mean ± SDs; *n* = 3 (***P < 0.001, ****P < 0.0001, two-tailed Student's *t*-test).

We confirmed the high expression of IDH3α, IDH3β and IDH3γ in quiescent compared with cycling Mel-RM and A375 cells using immunoblotting (Figure 5C). In contrast, there were no significant differences in the expression of IDH1 and IDH2, two NADP (+)-dependent IDH enzymes, between quiescent and cycling Mel-RM and A375 cells (Figure S16). Indeed, IDH3 but not IDH1 and IDH2 enzymatic activity was higher in quiescent than cycling cells (Figure 7A, B). Moreover, quiescent Mel-RM and A375 cells displayed higher levels of α-KG and increased NADH/NAD^+^ ratio, the specific by-product of isocitrate conversion by IDH3, and lower levels of HIF-1α expression than cycling Mel-RM cells, implicating low levels of glycolysis activity (Figure 7C-E).

To consolidate the functional significance of IDH3 in metabolic switching towards OXPHOS in quiescent cells, we introduced an inducible shRNA system to conditionally knock down the catalytic subunit of IDH3, IDH3α, in response to Dox, into dually genome-edited Mel-RM and A375cells (Figure 7F). Induced knockdown of IDH3α caused reduction in the levels of α-KG, NADH/NAD^+^ ratio, diminished the increase in OCR and mitochondrial oxidative stress, partially rescued the decrease in the ECAR and lactate and reduced the cell viability in quiescent cells (Figure 7G-M). Conversely, it did not significantly affect the ECAR and cell viability in cycling Mel-RM and A375 cells (Figure 7K and M). Taken together, these results demonstrate that the relatively high expression of IDH3 subunits plays an important role in the increased OXPHOS and promotion of cell survival in quiescent cells. In accordance, serum starvation triggered moderate upregulation of IDH3α, IDH3β and IDH3γ (Figure 7N), whereas siRNA knockdown of IDH3α, similar to inhibition of c-Myc and OXPHOS, reduced the accumulation of the quiescent population and enhanced induction of cell death triggered by serum starvation in MM200 cells (Figure 7O, P).

## Discussion

We have taken the advantage of high and low expression of p27 and Ki67, respectively, in quiescent cells and demonstrated that dual genome-editing to label endogenous p27 and Ki67 with differentiable fluorescent proteins is a stringent approach for the identification, purification and analysis of quiescent melanoma cells 27. Since the levels of p27 and Ki67 change inversely and progressively as cells exit and re-enter the cell cycle 12, 13, the combination of high p27 and low Ki67 as a marker ensures the rigor of purification of quiescent cancer cells. The quiescent state of isolated p27^high^Ki67^low^ cells was substantiated by low content of nucleic acid, diminished cell cycle progression signal pathways and downregulated expression of cell cycle progression genes. Of note, a small population (~5%) of isolated p27^high^Ki67^low^ cells expressed relatively high levels of cell cycle progression genes, which might represent cells at different depth of quiescence or those in the cell cycle but were nonetheless misguided into the quiescent population as a result of non-specific leakage during the isolation process 2, 30. Although further delineation of the origin and biological properties of this population is warranted, our results have established an experimental system that allows for isolating quiescent cancer cells with high purity (~95%). This application exploits the constitutive expression of cell cycle indicators that are regulated by diverse mechanisms, contrasting previous approaches using exogenous fluorescent probes whose regulation lacks the full endogenous intricacies 15, 45, 46. Moreover, this system is readily applicable to other types of cells apart from melanoma cells that were used in this study, and is potentially useful for generation of differentiable fluorescence-labelled p27 and Ki67 animal models for understanding cellular quiescence-related pathophysiological processes such as tissue-specific growth control and regeneration and immunological memory 47-49. In particular, the system provides a potent tool for investigation of the role of cellular quiescence in cancer initiation, responses to treatment, recurrence, and metastasis.

An important finding of this study was that the OXPHOS pathway was significantly enriched and the increased OXPHOS activity was the major metabolic program that supported quiescent cell survival. This is consistent with the notion that quiescent cells are not hypometabolic and suggests that some, if not all, of previously described OXPHOS-dependent cancer cells are in quiescent state 37, 50, 51. Of interest, while chemotherapeutic and molecularly targeted drugs act predominantly on cycling cells through induction of cell death or inhibition of cell cycle progression 52, 53, cancer cells surviving these treatments are frequently reliant more on OXPHOS for energy supply 37, 54. For example, melanoma cells resistant to MAPK inhibition display increased OXPHOS activity and are susceptible to treatment with OXPHOS inhibitors 37, whereas colon cancer cells resistant to 5-fluorouracil and glioma cells resistant to temozolomide are also dependent on OXPHOS and the addition of OXPHOS inhibitors enhances the efficacy of these drugs 53, 55. This metabolic phenotype in drug-resistant cancer cells may result from therapy-selected pre-existing subsets of cancer cells that are constitutively dependent on OXPHOS or is likely a consequence of therapy-induced metabolic switch towards OXPHOS 56, 57. Irrespectively, the increased OXPHOS is conceivably associated with cellular quiescence 58. In support, cancer stem-like cells that are commonly in quiescent state rely on OXPHOS for energy production and are refractory to therapeutic drugs 59. Moreover, slow-cycling cancer cells that share many biological properties with cancer cells in quiescence are also dependent on OXPHOS 60.

To our surprise, the proto-oncoprotein c-Myc was expressed at relatively high levels in quiescent melanoma cells. This is in stark contrast to the well-established role of c-Myc in promoting malignant cell cycle progression 61, 62. Indeed, the expression of c-Myc is frequently upregulated in many cancers including melanoma and overexpression of c-Myc promotes melanoma tumorigenicity and metastasis 63-65. Moreover, c-Myc has previously been shown to be downregulated in cells undergoing quiescence 63, 64, 66. This paradox is possibly caused by different purities of quiescent cell populations examined by different studies 67, 68. While we examined a highly purified quiescent cell population, many others looked at mixed cell populations after induction of quiescence by serum starvation, contact inhibition or treatment with cytotoxic or cytostatic agents 69-71. Notwithstanding the underlying reasons for the expression differences observed for c-Myc, our results showed that, consistent with the reduction in cell cycle progression gene expression, the association of c-Myc with the promoters of these genes was reduced, indicating that c-Myc is indeed unable to drive cell cycle progression even its expression is increased in quiescent cells. In contrast, the binding of c-Myc to its OXPHOS target genes was increased and c-Myc knockdown reduced OXPHOS gene expression, indicating that c-Myc preferentially drives transcriptional activation of these genes through selective occupancy of their promoters. It is therefore conceivable that while c-Myc plays predominantly a role in promoting cell proliferation and tumorigenicity in cycling cancer cells 72, it primarily supports survival of quiescent cancer cell through driving OXPHOS.

Specific occupancy of target promoters by c-Myc has been previously documented 40, 41, 73, although the regulatory mechanisms responsible remain to be fully defined. It has been proposed that the long noncoding RNA EPIC1 may specifically affect c-Myc occupancy of canonical MYC-MAX binding sites or function as a guide RNA to facilitate MYC-MAX regulation of selective targets through directly binding to target promoters 40. Whether a similar mechanism is involved in regulating the selective occupancy of OXPHOS gene promoters by c-Myc in quiescent cells remains to be determined. It is nevertheless known that c-Myc as a master regulator of metabolism plays an important role in metabolic adaptation to various cellular stress 74, 75. Similarly, how c-Myc is transcriptionally upregulated in quiescent cancer cells needs further investigation. A large array of transcription factors including Sp1 and nuclear factor I (NF-I) are known to activate transcription of *MYC* in response to diverse stimuli 76, 77.

Among OXPHOS genes upregulated by c-Myc in quiescent cells were those encoding subunits of IDH3 that catalyses the conversion of isocitrate to α-KG in an NAD (+)-dependent manner 43. An important function of α-KG is its role as an electron donor to PHDs, which enables VHL-mediated HIF-1α polyubiquitination and subsequent proteasomal degradation, leading to reduction in glycolysis activity 44. In accordance, knockdown of IDH3α, the catalytic subunit of IDH3, decreased the levels of α-KG, NADH, and HIF-1α, diminished the increase in the OCR and mitochondrial oxidative stress, and abolished the decrease in the ECAR and lactate in quiescent cells, consolidating the role of IDH3 in the metabolic switch towards OXPHOS in quiescent melanoma cells. Of note, the levels of the other IDHs, IDH1 and IDH2, which act in the cytoplasm and mitochondrion, respectively, as NADP (+)-dependent enzymes that similarly metabolize isocitrate to α-KG 42, were unaltered, corroborating the functional specificity of IDH3 in the metabolic switch in quiescent melanoma cells. c-Myc has previously been shown to repress the activity of IDH1 and thus results in downregulation of α-KG and upregulation of HIF-1α leading to increased glycolytic activity 78. Indeed, c-Myc and HIF-1α have long been known to interact, either directly or indirectly, in a cooperative or antagonistic manner 79-81. Therefore, the effects of c-Myc on IDH enzymes appear to be broadly important in regulating crosstalk between OXPHOS and glycolysis but the actions occur in a highly context-dependent manner.

Cellular quiescence renders cancer cells resistant to cell death and refractory to therapeutic drugs, and thus underlies cancer recurrence and metastasis even after initial responses to treatment 5. It is therefore long appreciated that curative cancer treatment requires therapies that either sustain the dormant state or effectively kill quiescent cells 6. However, making cancer cells quiescent indefinitely needs life-long treatment and thus is limited in practical terms 82, while conversely driving cancer cells out of quiescence to regain sensitivity to cytotoxic therapies may risk worsening patient outcomes should the therapies fail 6. Such drawbacks suggest that the optimal approach would be to eradicate quiescent cancer cells. Our results identify a c-Myc-driven IDH3-mediated metabolic switch toward OXPHOS as a critical mechanism supporting the survival of quiescent cancer cells, suggesting that targeting OXPHOS in combination with therapeutic agents that predominantly effect on cycling cells as a potential strategy towards curative treatment of cancer 83. OXPHOS inhibitors are emerging as promising agents in cancer therapy 84.

## Supplementary Material

Supplementary figures and tables.

## Abbreviations

3-D: Three-dimensional
AGRF: Australian Genome Research Facility
bcRNA-seq: bulk cell RNA-sequencing
BMIF: Biomedicain Imaging Facility
CDK: cyclin-dependent kinase
ChIP: Chromatin Immunoprecipitation
DAPI: 2-(4-Amidinophenyl)-6-indolecarbamidine dihydrochloride
DNP: 2,4-dinitrophenol
ECAR: extracellular acidification rate
FACS: fluorescence-activated cell sorting
GFP: green fluorescence protein
GSEA: gene set enrichment analysis
IDH: isocitric dehydrogenase
NF-I: nuclear factor I
OCR: oxygen consumption rate
OXPHOS: oxidative phosphorylation
PHD: prolyl hydroxylases
Rb: retinoblastoma
ROS: Reactive oxygen species
RT: room temperature
scRNA-seq: single cell RNA-sequencing
sgRNA: Single-guide RNA
STR: short tandem repeat
α-KG: α-ketoglutarate
