# Visualization of endogenous p27 and Ki67 reveals the importance of a c-Myc-driven metabolic switch in promoting survival of quiescent cancer cells: Erratum

**DOI:** 10.7150/thno.108351

**Published:** 2024-12-15

**Authors:** Ting La, Song Chen, Tao Guo, Xiao Hong Zhao, Liu Teng, Dandan Li, Michael Carnell, Yuan Yuan Zhang, Yu Chen Feng, Nicole Cole, Alexandra C. Brown, Didi Zhang, Qihan Dong, Jenny Y. Wang, Huixia Cao, Tao Liu, Rick F. Thorne, Feng-Min Shao, Xu Dong Zhang, Lei Jin

**Affiliations:** 1 School of Biomedical Sciences and Pharmacy, The University of Newcastle, NSW, 2308, Australia.; 2 Translational Research Institute, Henan Provincial People's Hospital and People's Hospital of Zhengzhou University, Henan Provincial and Zhengzhou City Key laboratory of Long Non-coding RNA and Cancer Metabolism, Henan, 450053, China.; 3 Centre for Excellence in Molecular Plant Sciences, Chinese Academy of Sciences, Shanghai, 200032, China.; 4 Department of Pulmonary and Critical Care Medicine, Henan Provincial People's Hospital, Zhengzhou University People's Hospital, Henan 450003, China.; 5 Biomedical Imaging Facility, University of New South Wales, NSW, 2052, Australia.; 6 Department of Orthopaedics, John Hunter Hospital, Hunter New England Health, NSW, 2305, Australia.; 7 Central Clinical School and Charles Perkins Centre, The University of Sydney, Sydney 2006, Australia.; 8 Children's Cancer Institute Australia for Medical Research, University of New South Wales, NSW 2750, Australia.; 9 Department of Nephrology, Henan Provincial People's Hospital, Zhengzhou University People's Hospital, Henan Provincial Clinical Research Canter for Kidney Disease, Henan 450003, China.

The authors regret that the original version of our paper, unfortunately, contained an incorrect picture in Figure 1F, where an incorrect image for Cyclin D1 of A375.DE cells was mistakenly used. The correct version of Figure 1F is shown below.

The correction made in this erratum does not affect the original data and conclusions. The authors apologize for any inconvenience that the errors may have caused.

## Figures and Tables

**Figure 1:**
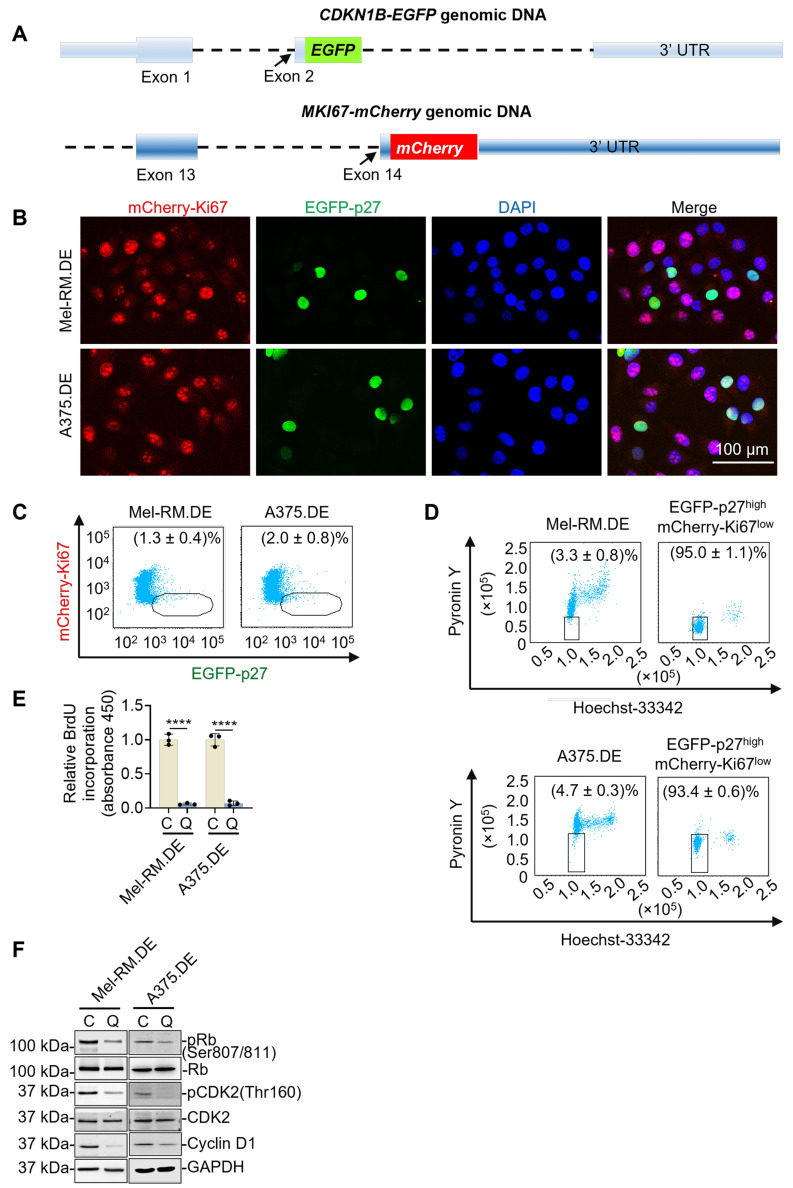
Correct image.

