# Supplementary material for: Visualization of endogenous p27 and Ki67 reveals the importance of a c-Myc-driven metabolic switch in promoting survival of quiescent cancer cells
Source: Theranostics. 2021 Sep 21;11(19):9605–22. doi: 10.7150/thno.63763 (PMC8490506; doi:10.7150/thno.63763)
Supplement: Supplementary file 1 — Supplementary figures and tables. [file thnov11p9605s1.pdf]

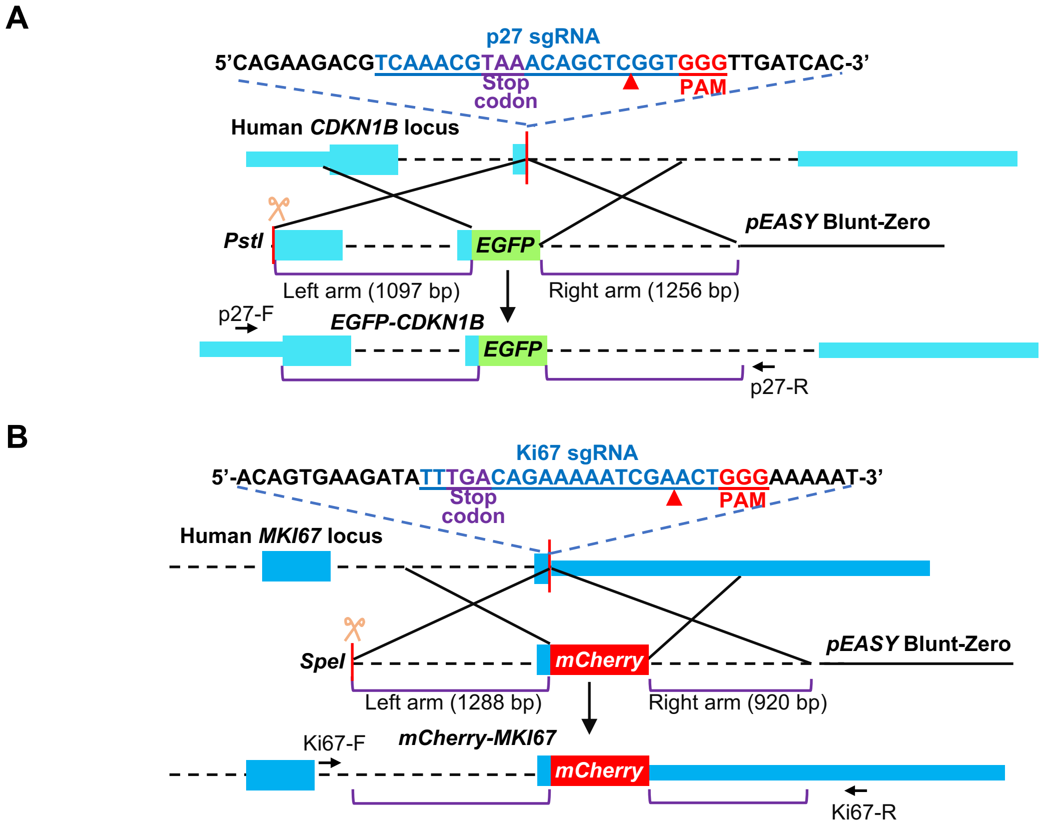


**Figure S1. Schematics of the targeting strategy to insert *EGFP* and *mCherry* sequences into the endogenous *CDKN1B* and *MKI67* loci, respectively, via homologous recombination.** The sgRNA sequences (underlined) were designed to span across the stop codons (TAA or TGA) of the endogenous *CDKN1B* or *MKI67,* respectively. Protospacer adjacent motif (PAM) sequences were marked in red. The restriction enzyme *PstI* or *SpeI* were used to linearize the targeting (donor) vectors. The PCR primers (p27-F + p27-R and Ki67-F + Ki67-R) used for genotyping are indicated with horizontal arrows. The left and right homology arms were marked with purple lines. p27-F: Forward primer for EGFP-p27 genotyping; p27-R: Reverse primer for EGFP-p27 genotyping; Ki67-F: Forward primer for mCherry-Ki67 genotyping; Ki67-R: Reverse primer for mCherry-Ki67 genotyping.

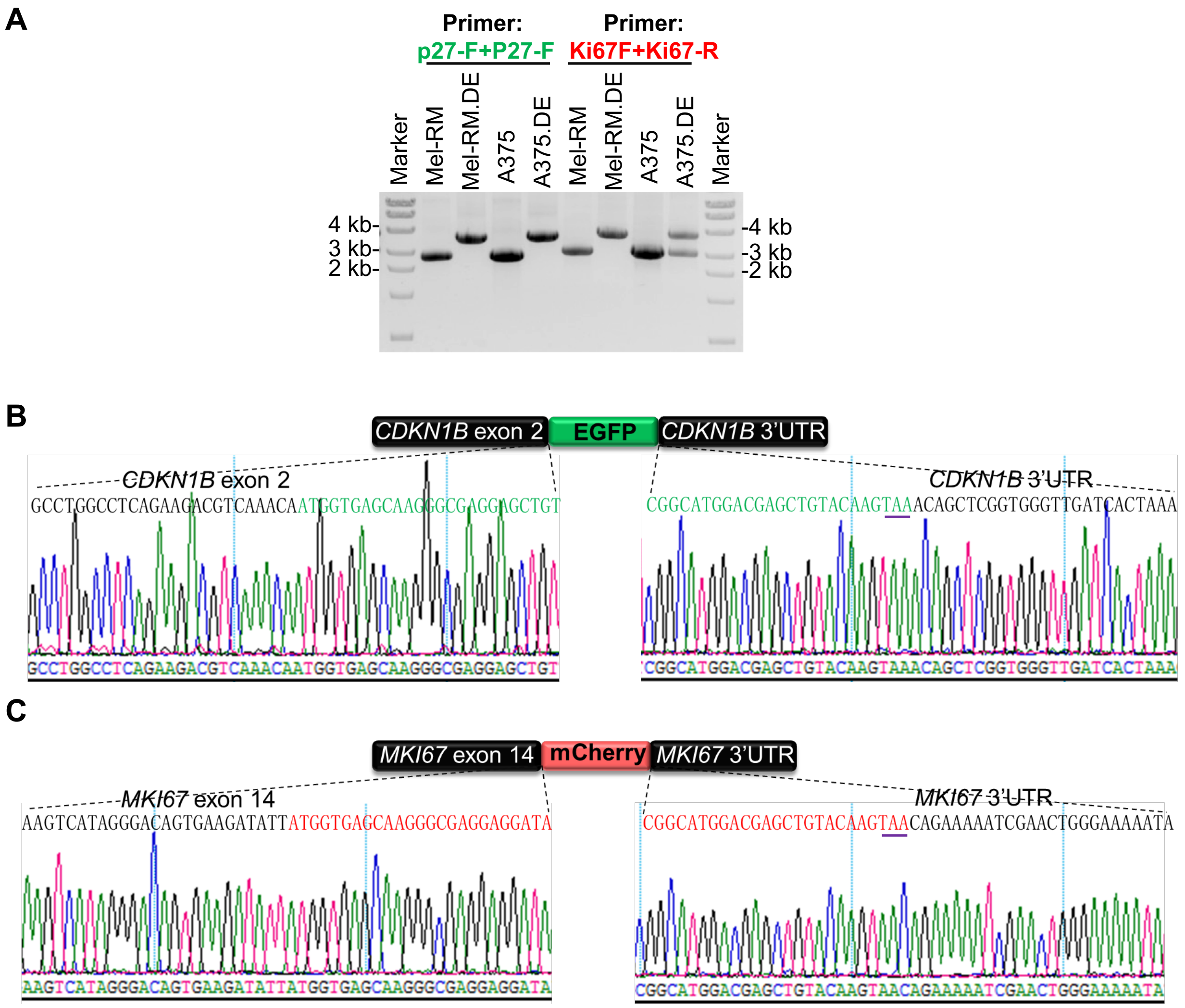


**Figure S2. PCR and Sanger sequencing to confirm the accurate insertion of the *EGFP* and *mCherry* sequences at the C-termini of *CDKN1B* and *MKI67*, respectively.** (**A**) PCR analysis of the targeted alleles containing the *EGFP* and *mCherry* sequences in dually edited Mel-RM (Mel-RM.DE) and A375 (A375.DE) cells. $n$ = 3. The primers used were illustrated in Figure S1. (**B and C**) Sanger sequencing demonstrated the precise integration of *EGFP* (B) and *mCherry* (C) at the C-termini of *CDKN1B* (B) and *MKI67* (C), respectively. The stop codons are underlined.

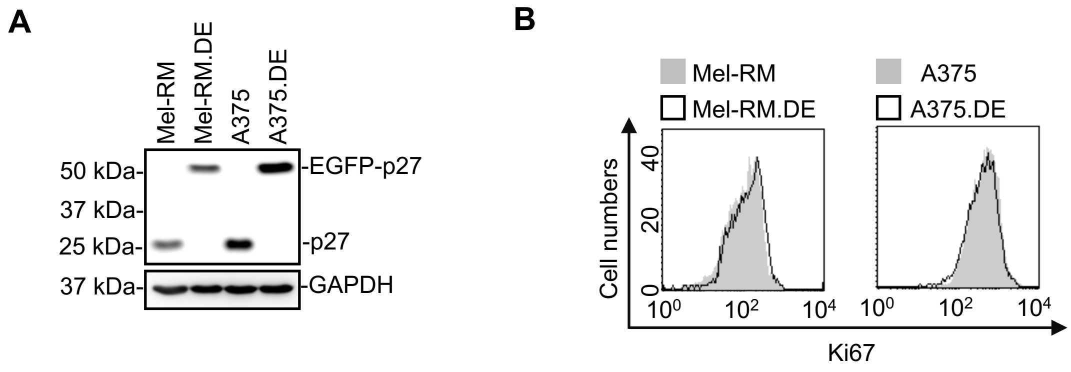


**Figure S3. The levels of p27 and Ki67 expression remain unaltered in dually edited cells.** (**A**) Whole cell lysates from parental Mel-RM and A375 and dually edited Mel-RM (Mel-RM.DE) and A375 (A375.DE) cells were analysed using Western blotting. *n* = 3. (**B**) Representative flowcytometric histograms showing the dually edited cells and corresponding parental cells expressed similar levels of Ki67 identified using an APC conjugated anti-Ki-67 antibody. *n* = 3.

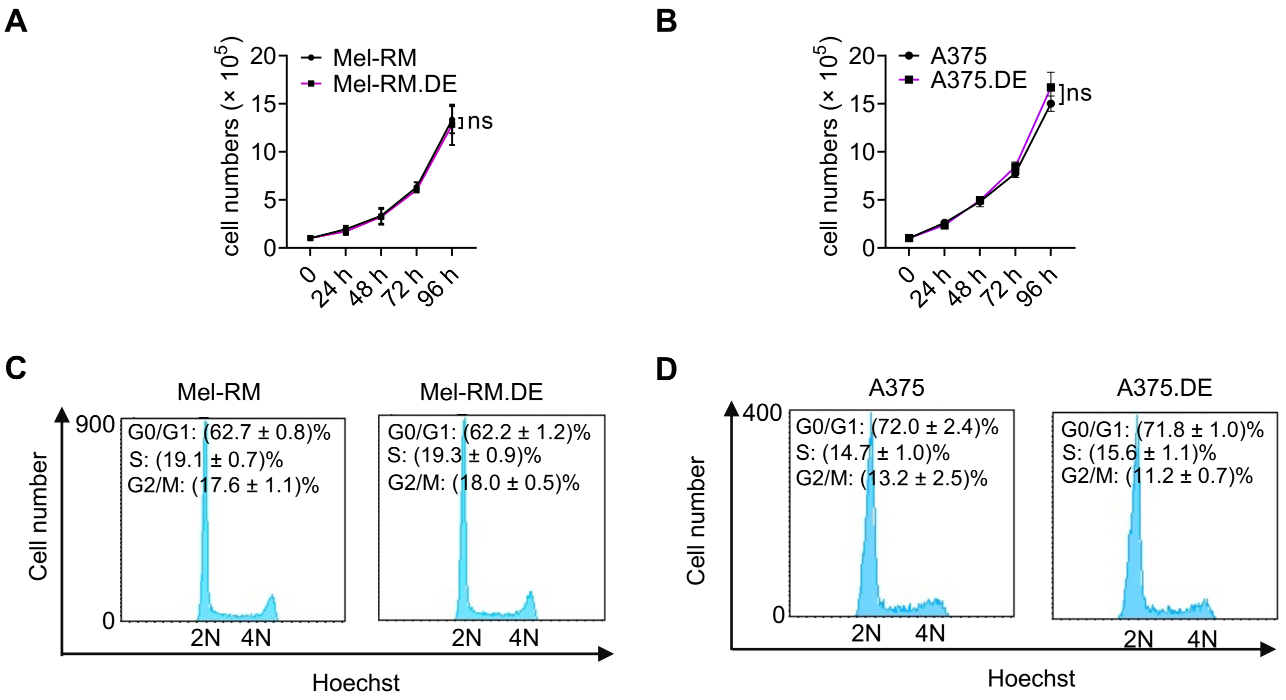


**Figure S4. The proliferation rate and cell cycle distribution remain unaltered in dually edited cells.** (**A and B**) Viable parental Mel-RM and A375 and dually edited Mel-RM (Mel-RM.DE) and A375 (A375.DE) cells were counted using an automated cell counter 24, 48, 72, and 96 h after seeding. Values are mean ± SDs, $n = 3$ ($P > 0.05$, two-tailed Student's *t*-test ). (**C and D**) Mel-RM, Mel-RM.DE, A375 and A375.DE cells were subjected to Propidium iodide (PI) analysis of cell cycle distribution using flow cytometer. Values are mean ± SDs; $n = 3$.

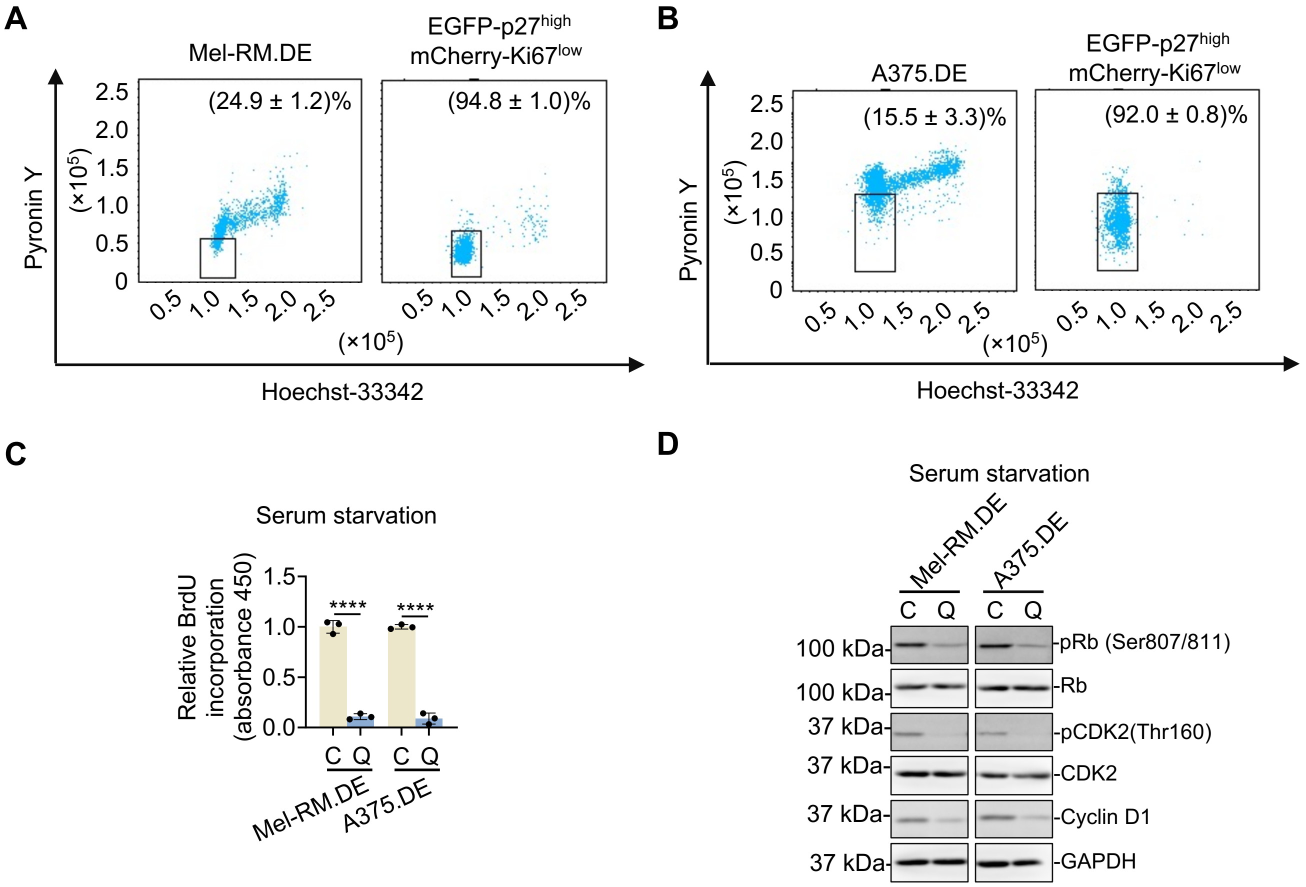


**Figure S5. EGFP-p27$^{high}$mCherry-Ki67$^{low}$ cells display quiescent characteristics.** (**A and B**) Representative flowcytometry dot plots of EGFP-p27$^{high}$mCherry-Ki67$^{low}$ cells isolated from dually edited Mel-RM (Mel-RM.DE; A) and A375 (A375.DE ;B) cells with DNA and RNA labelled using Hoechst-33342 and Pyronin Y staining, respectively. Values are mean ± SDs; *n* = 3. (**C**) The DNA synthesis activity was not detected in EGFP-p27$^{high}$mCherry-Ki67$^{low}$ [quiescent cells (Q)] cells, but was readily detectable in the other cells [cycling cells (C)] isolated from Mel-RM.DE and A375.DE cells upon serum starvation as shown in BrdU incorporation assays. Values are mean ± SDs; *n* = 3 ( ****$P$ < 0.0001, two-tailed Student's *t*-test). (**D**) Whole cell lysates from EGFP-p27$^{high}$mCherry-Ki67$^{low}$ quiescent (Q) and cycling cells isolated from Mel-RM.DE and A375.DE cells undergoing serum starvation were subjected to Western blotting. *n* = 3.

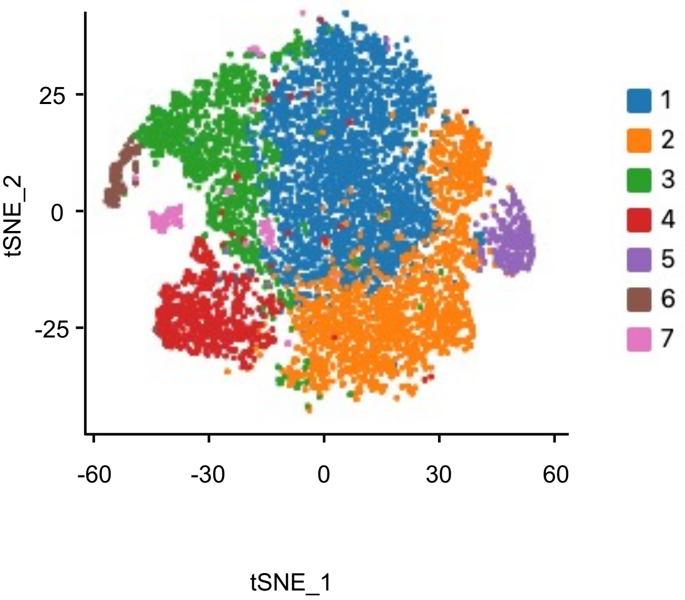


**Figure S6.** t-Distributed stochastic neighbor embedding (t-SNE) visualization of transcriptomes of 7554 single EGFP-p27$^{high}$mCherry-Ki67$^{low}$ quiescent cells isolated from dually edited Mel-RM cells upon serum starvation. Cells are colored by their associated clusters (1-7).

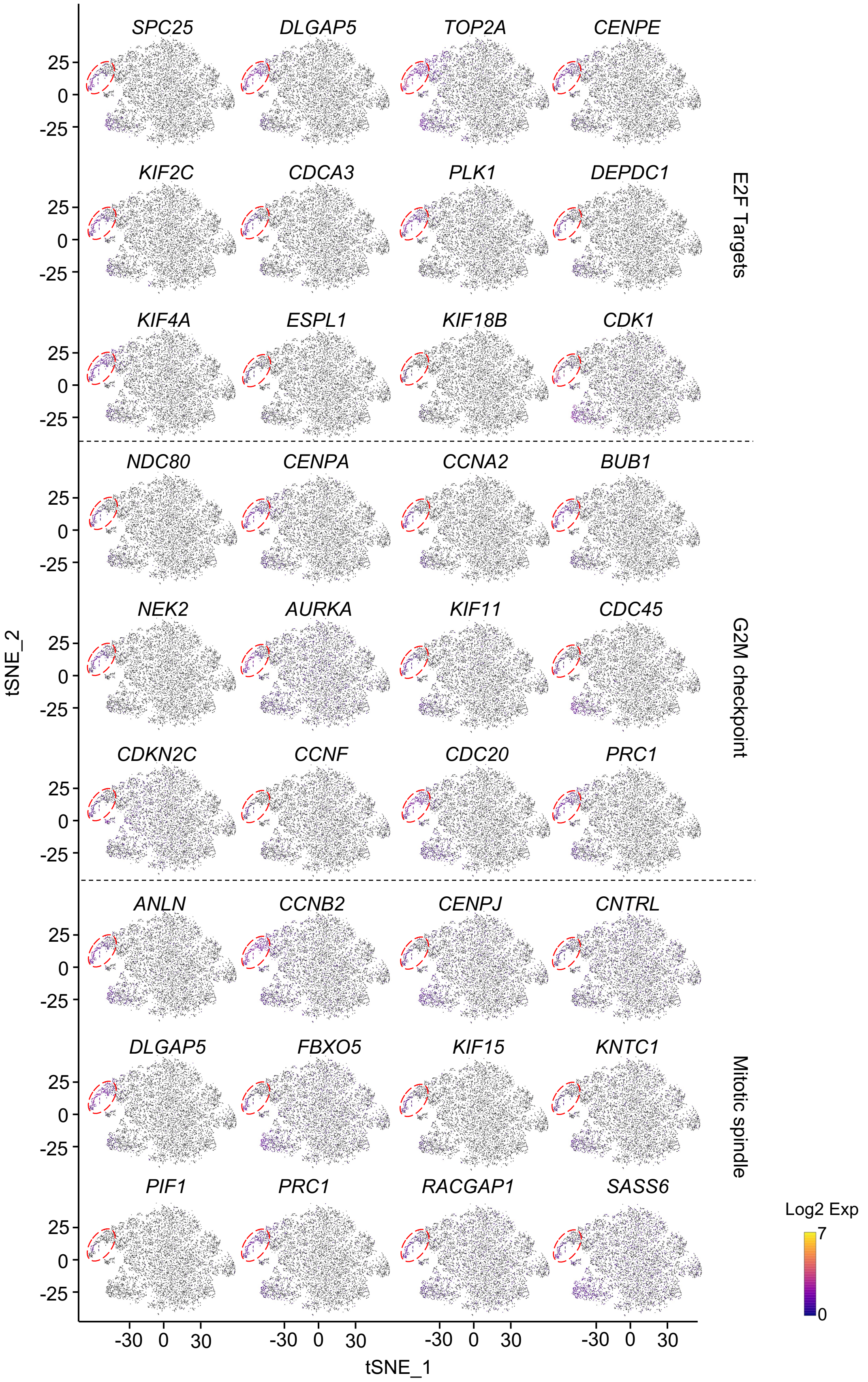


**Figure S7. EGFP-p27^high^mCherry-Ki67^low^ cells express no or low levels of representative E2F, G2M checkpoint and mitotic spindle assembly pathway genes.** t-SNE plots of single cell RNA-sequencing data showing that representative genes of the E2F, G2M checkpoint and mitotic spindle assembly pathways were not expressed or detected at low levels in the majority of EGFP-p27$^{high}$mCherry-Ki67$^{low}$ cells isolated from dually edited Mel-RM cells undergoing serum starvation. A small population of cells that expressed relatively high levels of these genes are marked by ellipses.

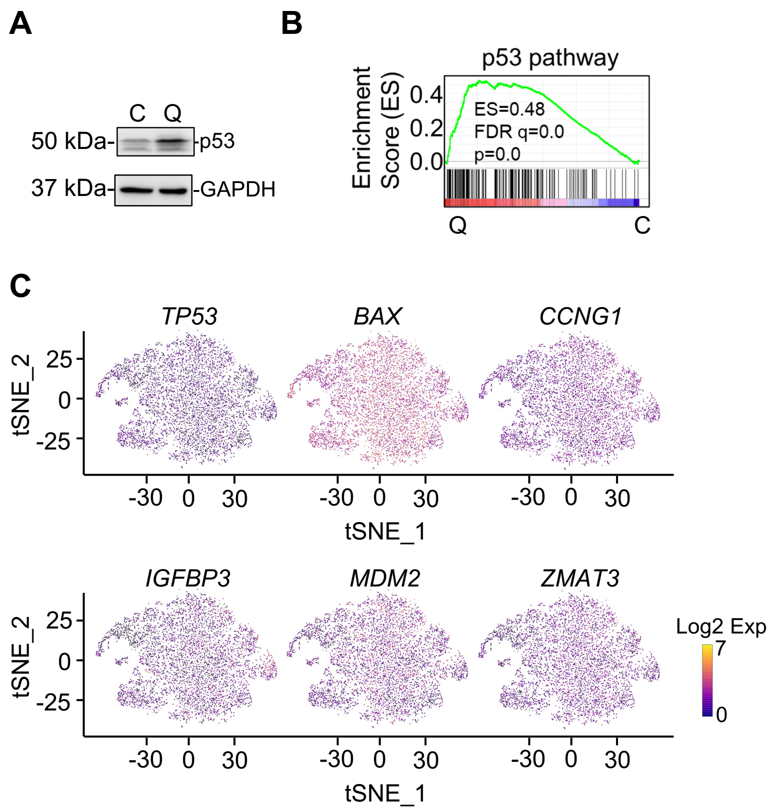


**Figure S8. The p53 pathway is enriched in quiescent cells.** (**A**) Whole cell lysates from EGFP-p27$^{high}$mCherry-Ki67$^{low}$ quiescent (Q) and cycling (C) cells isolated from dually edited Mel-RM cells undergoing serum starvation were analysed using Western blotting. $n$ = 3. (**B**) A GSEA plot of bulk cell RNA-sequencing (bcRNA-seq) data showing that the p53 pathway was enriched in EGFP-p27$^{high}$mCherry-Ki67$^{low}$ quiescent (Q) compared with cycling (C) cells isolated from dually edited Mel-RM cells undergoing serum starvation. FDR, false-discovery rate; ES, enrichment score. $n$ = 2 biological repeats. (**C**) t-SNE plots of single cell RNA-sequencing (scRNA-seq) data showing that *TP53* and representative p53 target genes were expressed in EGFP-p27$^{high}$mCherry-Ki67$^{low}$ quiescent cells isolated from dually edited Mel-RM cells undergoing serum starvation.

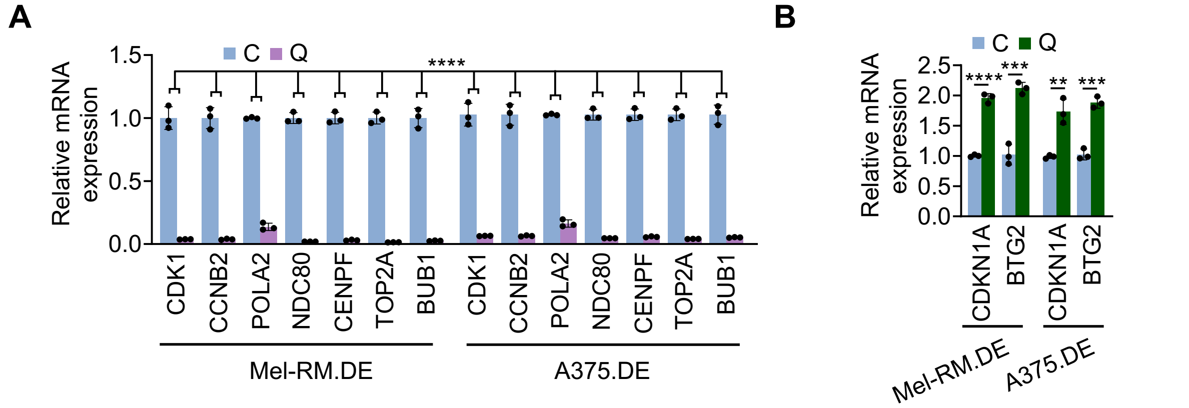


**Figure S9. Validation of the changes in representative E2F, G2M progression, mitotic spindle assembly and p53 pathway genes in quiescent compared with cycling cells.** Total RNA from EGFP-p27$^{high}$mCherry-Ki67$^{low}$ quiescent (Q) and cycling (C) cells isolated from dually edited Mel-RM (Mel-RM.DE) and A375 (A375.DE) cells undergoing serum starvation were subjected to qPCR analysis of the indicated representative E2F, G2M progression, mitotic spindle assembly pathway genes (A) and p53 pathway genes (B) . Values are mean ± SDs; $n$ = 3 (**$P < 0.01$; ***$P < 0.001$; ****$P < 0.0001$, two-tailed Student's $t$-test ).

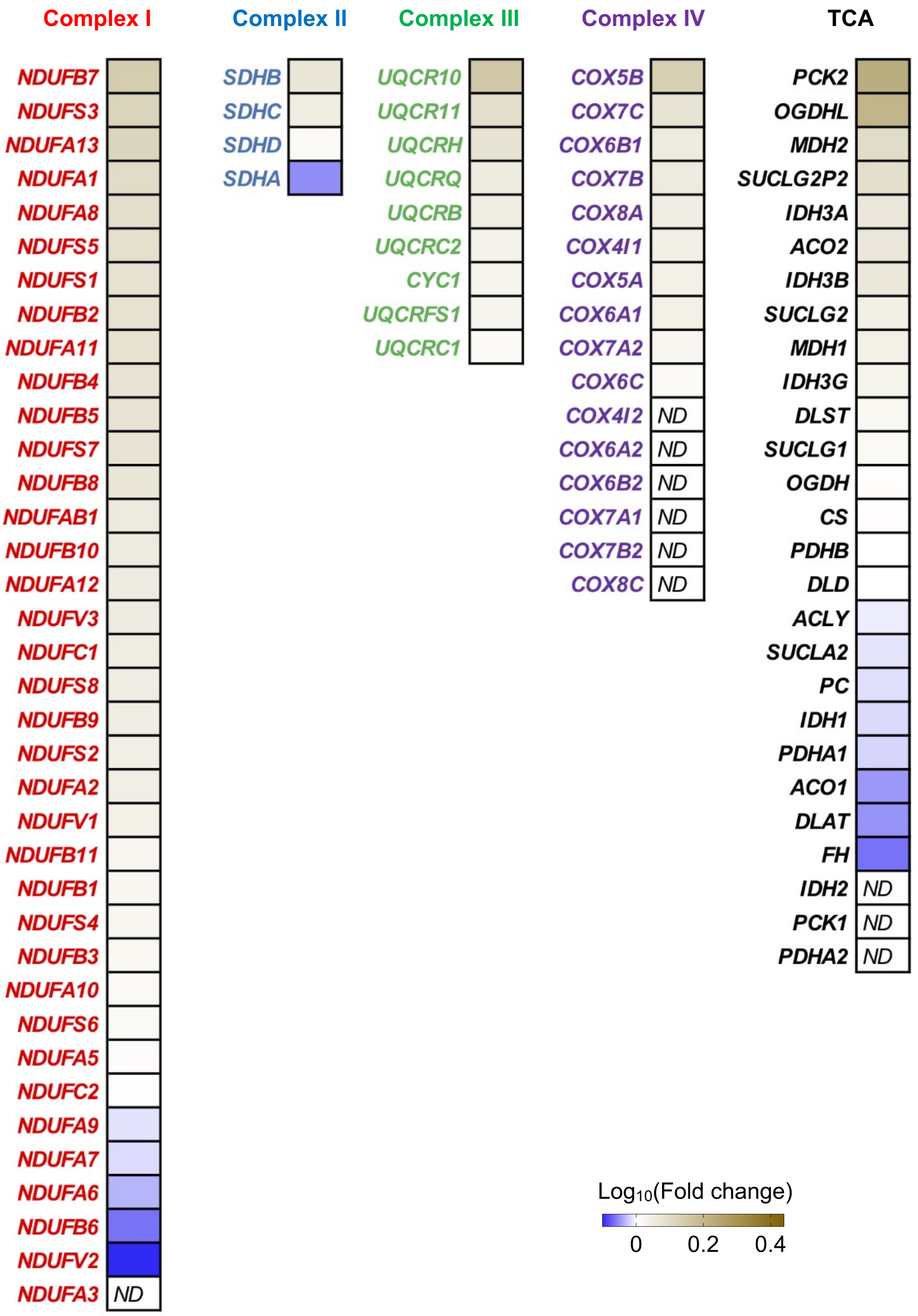


**Figure S10. Upregulation of OXPHOS and TCA genes in quiescent cells.** Log10-transformed fold gene expression changes derived from bulk cell RNA-sequencing of EGFP-p27$^{high}$mCherry-Ki67$^{low}$ quiescent and cycling cells isolated from dually edited Mel-RM cells undergoing serum starvation were subjected to hierarchical clustering and displayed in the thumbnail-dendogram format. *n* = 2 biological repeats. *ND*: not detectable by RNA-seq.

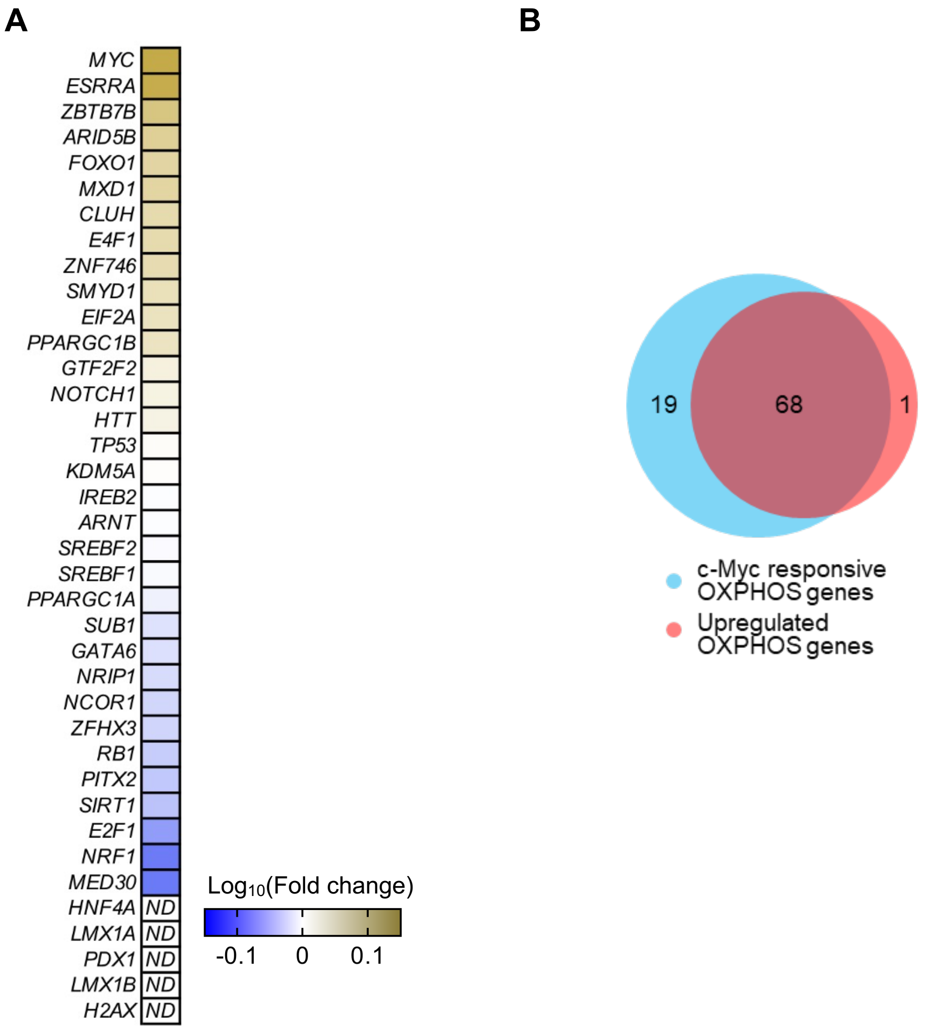


**Figure S11. *MYC* and c-Myc-responsive OXPHOS genes are expressed at higher levels in quiescent compared with cycling cells. (A)** Log10-transformed fold gene expression changes derived from bulk cell RNA-sequencing of EGFP-p27$^{high}$mCherry-Ki67$^{low}$ quiescent and cycling cells isolated from dually edited Mel-RM cells undergoing serum starvation were subjected to hierarchical clustering and displayed in the thumbnail-dendogram format. *MYC* was the most prominently upregulated gene among those encoding transcription factors that regulate OXPHOS gene expression in EGFP-p27$^{high}$mCherry-Ki67$^{low}$ quiescent compared to cycling cells. $n = 2$ biological repeats**. (B)** Venn diagram illustrating that almost all OXPHOS genes that were upregulated in EGFP-p27$^{high}$mCherry-Ki67$^{low}$ quiescent compared to cycling cells isolated from dually edited Mel-RM cells undergoing serum starvation were those that c-Myc-responsive genes. c-Myc responsive genes were defined according to the Encyclopedia of DNA Elements (ENCODE) transcription factor binding sites (TFBS) chromatin immunoprecipitation (ChIP)-seq data.

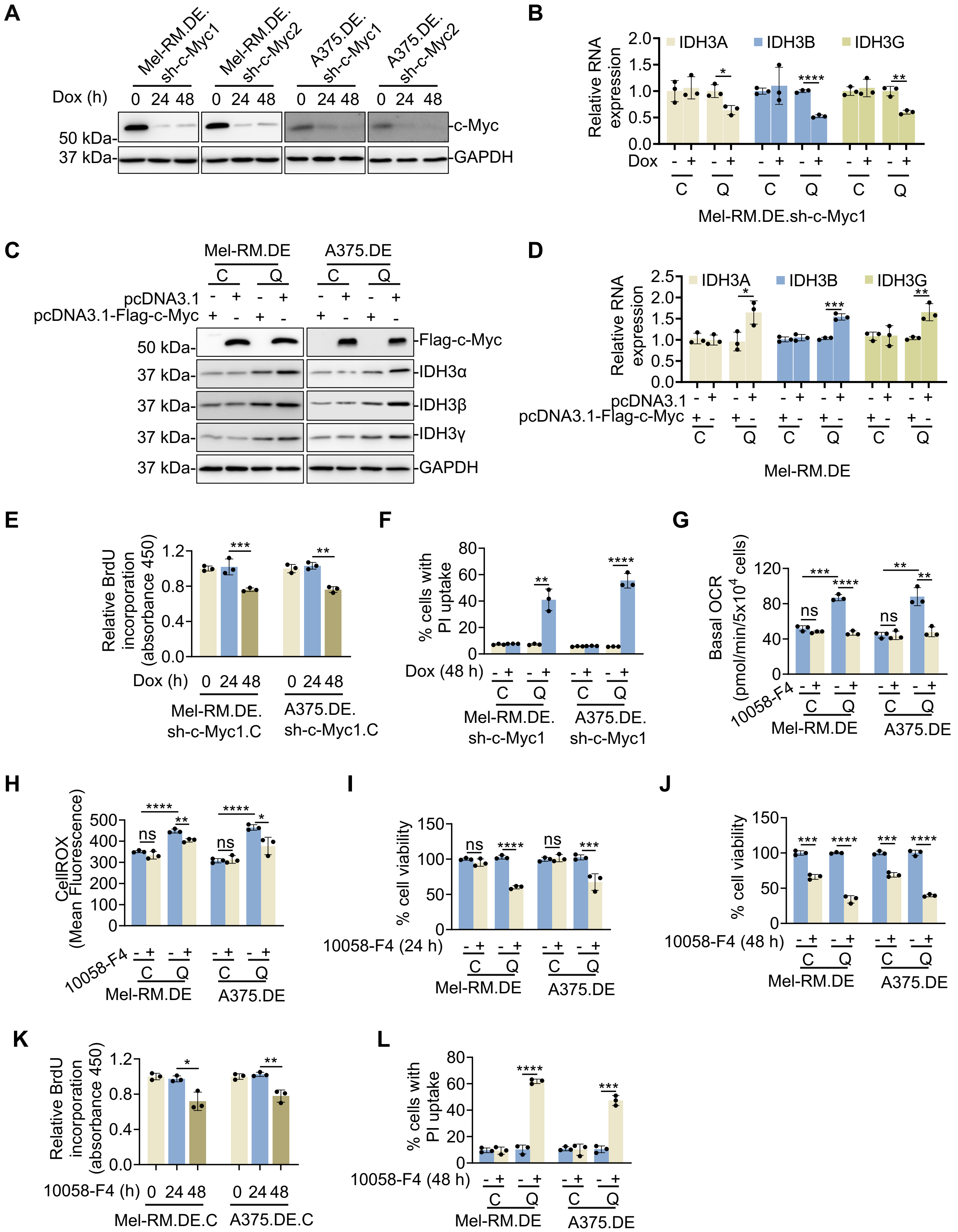

A
Mel-RM.DE. sh-c-Myc1
Mel-RM.DE. sh-c-Myc2
A375.DE. sh-c-Myc1
A375.DE. sh-c-Myc2
Dox (h) 0 24 48
50 kDa-
37 kDa-
-c-Myc
-GAPDH
B
Relative RNA expression
IDH3A
IDH3B
IDH3G
Dox
C Q
Mel-RM.DE.sh-c-Myc1
C
Mel-RM.DE
A375.DE
pcDNA3.1
pcDNA3.1-Flag-c-Myc
-Flag-c-Myc
-IDH3α
-IDH3β
-IDH3γ
-GAPDH
D
Relative RNA expression
Mel-RM.DE
E
Relative BrdU incorporation (absorbance 450)
Dox (h)
Mel-RM.DE. sh-c-Myc1.C
A375.DE. sh-c-Myc1.C
F
% cells with PI uptake
Dox (48 h)
Mel-RM.DE. sh-c-Myc1
A375.DE. sh-c-Myc1
G
Basal OCR (pmol/min/5x10^4 cells)
10058-F4
Mel-RM.DE
A375.DE
H
CellROX (Mean Fluorescence)
I
% cell viability
10058-F4 (24 h)
J
% cell viability
10058-F4 (48 h)
K
Relative BrdU incorporation (absorbance 450)
10058-F4 (h)
Mel-RM.DE.C
A375.DE.C
L
% cells with PI uptake
10058-F4 (48 h)

**Figure S12. c-Myc drives OXPHOS and promotes cell survival in quiescent cells.** (**A**) Whole cell lysates from dually edited Mel-RM (Mel-RM.DE) and A375 (A375.DE) cells carrying an inducible c-Myc shRNA system with or without treatment with Doxycycline (Dox; 100ng/ml) for the indicated periods were subjected to Western blotting. *n* = 3. **(B)** Total RNA from EGFP-p27$^{high}$mCherry-Ki67$^{low}$ quiescent (Q) and cycling (C) cells isolated from Mel-RM.DE and A375.DE cells carrying an inducible c-Myc shRNA system with or without treatment with Doxycycline (Dox) for 24 h undergoing serum starvation were subjected to qPCR analysis. Values are mean ± SDs; *n* = 3 (**P* < 0.05; ***P* < 0.01 *****P* < 0.0001, two-tailed Student's *t*-test). **(C)** Whole cell lysates from EGFP-p27$^{high}$mCherry-Ki67$^{low}$ quiescent (Q) and cycling (C) cells isolated from Mel-RM.DE and A375.DE cells transfected with the indicated plasmids undergoing serum starvation were analyzed using Western blotting. *n* = 3. (**D**) Total RNA from EGFP-p27$^{high}$mCherry-Ki67$^{low}$ quiescent (Q) cells isolated from Mel-RM.DE cells transfected with the indicated plasmids undergoing serum starvation were subjected to qPCR analysis. Values are mean ± SDs; *n* = 3 (**P* < 0.05; ***P* < 0.01, ****P* < 0.001, two-tailed Student's *t*-test). **(E)** EGFP-p27$^{low}$mCherry-Ki67$^{high}$ cycling cells isolated from Mel-RM.DE (Mel-RM.DE.C) and A375.DE (A375.DE.C) cells carrying an inducible c-Myc shRNA system undergoing serum starvation were treated with Dox for the indicated periods were subjected to measurement of DNA synthesis using BrdU incorporation assays. The relative BrdU incorporation in cells of each cell line without Dox treatment was arbitrarily designated as 1, respectively. Values are mean ± SDs; *n* = 3 (***P* < 0.01; ****P* < 0.001, two-tailed Student's *t*-test). **(F)** EGFP-p27$^{high}$mCherry-Ki67$^{low}$ quiescent (Q) and cycling (C) cells isolated from Mel-RM.DE and A375.DE cells carrying an inducible c-Myc shRNA system undergoing serum starvation were treated with Dox for 48 h before cell death was measured using the PI uptake assay. Values are mean ± SDs; *n* = 3 (***P* < 0.01; *****P* < 0.0001, two-tailed Student's *t*-test ).(**G**) The basal oxygen consumption rate (OCR) measured using Seahorse XF analysis of EGFP-p27$^{high}$mCherry-Ki67$^{low}$ quiescent (Q) and cycling (C) cells isolated from Mel-RM.DE and A375.DE cells undergoing serum starvation for 96 h with or without treatment with 10058-F4 for 24 h. Values are mean ± SDs; *n* = 3 (***P* < 0.01; ****P* < 0.001; *****P* < 0.0001, two-tailed Student's *t*-test). (**H**) EGFP-p27$^{high}$mCherry-Ki67$^{low}$ quiescent (Q) and cycling (C) cells isolated from Mel-RM.DE and A375.DE cells undergoing serum starvation for 96 h with or without treatment with 10058-F4 for 24 h were subjected to CellROX analysis of oxidative stress levels. Values are mean ± SDs; *n* = 3 (**P* < 0.05; ***P* < 0.01; *****P* < 0.0001, two-tailed Student's *t*-test). (**I**) EGFP-p27$^{high}$mCherry-Ki67$^{low}$ quiescent (Q) and cycling (C) cells isolated from Mel-RM.DE and A375.DE cells undergoing serum starvation for 96 h with or without treatment with 10058-F4 for 24 h were subjected to CCK8 assays. Values are mean ± SDs; *n* = 3 (****P* < 0.001 *****P* < 0.0001, two-tailed Student's *t*-test). (**J**) EGFP-p27$^{high}$mCherry-Ki67$^{low}$ quiescent (Q) and cycling (C) cells isolated from Mel-RM.DE and A375.DE cells undergoing serum starvation in the presence or absence of 10058-F4 for 48 h were subjected to measurement of cell viability using CCK8 assays). Values are mean ± SDs; *n* = 3 (****P* < 0.001 *****P* < 0.0001, two-tailed Student's *t*-test. **(K)** EGFP-p27$^{low}$mCherry-Ki67$^{high}$ cycling cells isolated from Mel-RM.DE (Mel-RM.DE.C) and A375.DE (A375.DE.C) cells undergoing serum starvation were treated with 10058-F4 for the indicated periods were subjected to BrdU incorporation assays as described for (E). The relative BrdU incorporation in cells of each cell line without treatment was arbitrarily designated as 1, respectively. Values are mean ± SDs; *n* = 3 (**P* < 0.05 ***P* < 0.01, two-tailed Student's *t*-test). (**L**) EGFP-p27$^{high}$mCherry-Ki67$^{low}$ quiescent (Q) and cycling (C) cells isolated from Mel-RM.DE and A375.DE cells undergoing serum starvation in the presence or absence of 10058-F4 for 48 h was measured using the PI uptake assay. Values are mean ± SDs; *n* = 3 (***P* < 0.01; *****P* < 0.0001, two-tailed Student's *t*-test).

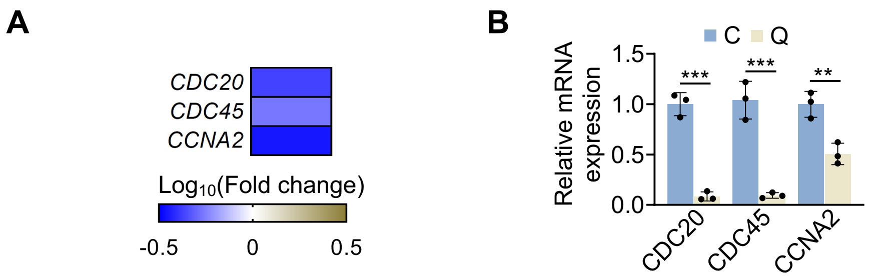


**Figure S13. c-Myc-responsive cell proliferation genes are expressed at low levels in quiescent cells.** (**A**) Log10-transformed fold gene expression changes derived from bulk cell RNA-sequencing of EGFP-p27$^{high}$mCherry-Ki67$^{low}$ quiescent and cycling cells isolated from dually edited Mel-RM cells undergoing serum starvation were subjected to hierarchical clustering and displayed in the thumbnail-dendogram format. $n$ = 2 biological repeats. (**B**) Total RNA from EGFP-p27$^{high}$mCherry-Ki67$^{low}$ quiescent (Q) and cycling (C) cells isolated from dually edited Mel-RM cells undergoing serum starvation were subjected to qPCR analysis. Values are mean ± SDs; $n$ = 3 (**$P$ < 0.01; ***$P$ < 0.001, two-tailed Student's $t$-test).

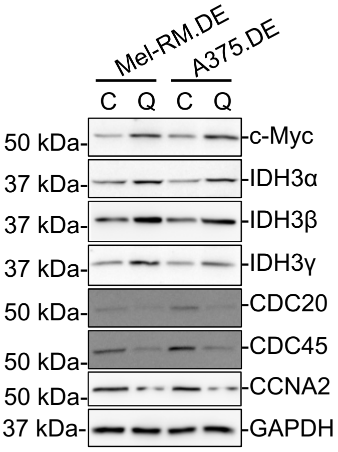


**Figure S14. EGFP-p27$^{high}$mCherry-Ki67$^{low}$ cells grown in 3-dimensional (3-D) cultures display higher levels of IDH3 and lower levels cell cycle progression genes.** Whole cell lysates from EGFP-p27$^{high}$mCherry-Ki67$^{low}$ quiescent (Q) and cycling (C) cells isolated from Mel-RM.DE and A375.DE cells grown in 3-D cultures subjected to serum starvation were subjected to Western blotting. $n$ = 3.

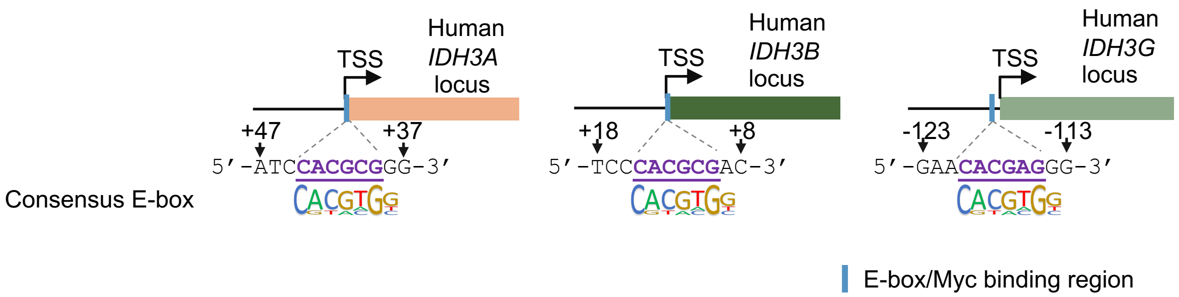


**Figure S15.** A schematic illustration of consensus E-box/c-Myc binding regions in the promoters of *IDN3A*, *IDH3B* and *IDH3G*.

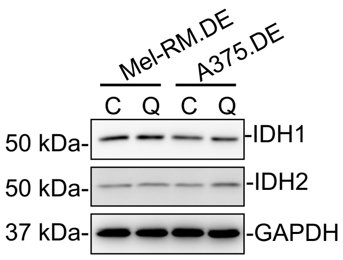


**Figure S16. IDH1 and IDH2 expression remain unaltered in quiescent cells.** Whole cell lysates from EGFP-p27$^{high}$mCherry-Ki67$^{low}$ quiescent (Q) and cycling (C) cells isolated from dually edited Mel-RM (Mel-RM.DE) and A375 (A375.DE) cells undergoing serum starvation were subjected to Western blotting. *n* = 3.

**Table S1. Oligonucleotides used for sgRNA plasmid construction and genotyping**

| Oligo | Sequence (5'-3') |
|---|---|
| p27 sgRNA-top | CACCGTCAAACGTAAACAGCTCG |
| p27 sgRNA-bottom | AAACACCGAGCTGTTTACGTTTG |
| Ki67 sgRNA-top | CACCGTTTGACAGAAAAATCGAA |
| Ki67 sgRNA-bottom | AAACAGTTCGATTTTTCTGTCAA |
| p27-F | TTTTTTTGAGAGTGCGAGAGAGG |
| p27-R | GGTCAAAGGCAAGTGGGAAATA |
| Ki67-F | GTGAGAGTTCCTGGTCAGTGGGG |
| Ki67-R | TGGATGACGCTGTGAGAACCCTA |

**Table S2. List of Reagents**

| Reagent | Catalogue No. | Company |
|---|---|---|
| 2,4-Dinitrophenol | 34334 | Sigma-Aldrich |
| 10058-F4 | F3680 | Sigma-Aldrich |
| IACS-010759 | 25867 | Cayman |
| Doxycycline hydrochloride | D3447 | Sigma-Aldrich |
| Puromycin dihydrochloride | P9620 | Sigma-Aldrich |
| bisBenzimide H 33258 | B2883-100mg | Sigma-Aldrich |
| Pyronin Y | P9172-1G | Sigma-Aldrich |
| Propidium iodide | P4170-1G | Sigma-Aldrich |
| pEASY® -Blunt Zero Cloning Kit | CB501-01 | TransGen Biotech |
| BrdU Cell Proliferation Assay Kit | #6813 | Cell Signaling |

**Table S3**. **List of antibodies**

| Antibody | Catalogue No. | Company |
|---|---|---|
| Phospho-Rb (Ser807/811) antibody | #9308 | Cell Signalling Technology (Beverly, MA) |
| Recombinant Anti-Rb antibody [EPR17512] | ab181616 | Abcam (Cambridge, United Kingdom) |
| HIF-1α Antibody (28b) | sc-13515 | Santa Cruz Biotechnology (Santa Cruz, CA) |
| Phospho-CDK2 (Thr160) Antibody | #2561 | Cell Signalling Technology (Beverly, MA) |
| Cdk2 (D-12) | sc-6248 | Santa Cruz Biotechnology (Santa Cruz, CA) |
| cyclin D1 Antibody (H-295) | sc-753 | Santa Cruz Biotechnology (Santa Cruz, CA) |
| p27 Kip1 (D69C12) XP® Rabbit mA | #3686 | Cell Signalling Technology (Beverly, MA) |
| GAPDH antibody (6C5) | sc-32233 | Santa Cruz Biotechnology (Santa Cruz, CA) |
| Anti-IDH3A antibody | ab58641 | Abcam (Cambridge, United Kingdom) |
| Anti-IDH3B antibody | ab247089 | Abcam (Cambridge, United Kingdom) |
| Anti-IDH3G antibody | ab224210 | Abcam (Cambridge, United Kingdom) |
| Recombinant Anti-SDHC antibody [EPR11035(B)] | ab155999 | Cell Signalling Technology (Beverly, MA) |
| Anti-NDUFV1 | ab203208 | Abcam (Cambridge, United Kingdom) |
| Recombinant Anti-IDH1 antibody [EPR12296] | ab172964 | Abcam (Cambridge, United Kingdom) |
| Recombinant Anti-IDH2 antibody [EPR7577] | ab131263 | Abcam (Cambridge, United Kingdom) |
| c-Myc Antibody | #9402 | Cell Signalling Technology (Beverly, MA) |
| APC anti-mouse Ki-67 antibody | 652406 | BioLegend (San Diego, CA) |

**Table S4. Oligonucleotides used for qPCR**

| Oligo | Sequence (5'-3') |
|---|---|
| CDK1 qPCR-F | GGTCAAGTGGTAGCCATGAAA |
| CDK1 qPCR-R | ATGTACTGACCAGGAGGGATAG |
| CCNB2 qPCR-F | GCAGTCCATAAACCCACATTTC |
| CCNB2 qPCR-R | GCAGAGCAGTAATCCCAACTA |
| POLA2 qPCR-F | ATTATCGAAAGCCAGGCATAGT |
| POLA2 qPCR-R | GAGAAACTTGACGGTGAGAGTA |
| NDC80 qPCR-F | TCTCTACAAGCTCCCTCTGTTA |
| NDC80 qPCR-R | CAAACTAAGGCTGCCACAATG |
| CENPF qPCR-F | GAGCTTGAAGGACAGCTTGA |
| CENPF qPCR-R | TCACTTGTGACTCCTTGACTTG |
| TOP2A qPCR-F | CTGCGGACAACAAACAAAGG |
| TOP2A qPCR-R | TTCGACCACCTGTCACTTTC |
| BUB1 qPCR-F | TTGCTCCTCCTGTTCCTTTG |
| BUB1 qPCR-R | CTGAACCATTCCCAGTGATGT |
| IDH3A qPCR-F | GCTGCCAAAGCACCTATT |
| IDH3A qPCR-R | TCAAGCCCATCTTGTTCTTATC |
| IDH3B qPCR-F | TTGGAAAGATTCATACCCCGAT |
| IDH3B qPCR-R | TGTTGTGCCGAGTCATATACCC |
| IDH3G qPCR-F | GTACCAGTGGACTTTGAAGAG |
| IDH3G qPCR-R | CGGACAATGAGGATGTCTATG |
| NDUFB7 qPCR-F | GATGCCAACCTTCCCGCCAGA |
| NDUFB7 qPCR-R | AGCTGTCACGCTTGCACTTGA |
| SDHB qPCR-F | TGATAAAGGATCTTGTTCCCGAT |
| SDHB qPCR -R | TTTCTCACGCTCTTCTATGGAC |
| UQCR10 qPCR-F | ATCTACGACCACATCAACGAGG |
| UQCR10 qPCR-R | CGTTTCTTGCCAACAGCGAAT |
| COX5B qPCR-F | CAATGGCTTCAAGGTTACTTCGC |
| COX5B qPCR-R | CTTTGCAGCCAGCATGATCTCC |
| c-Myc qPCR-F | TCCGTCCTCGGATTCTCTGCTCT |
| c-Myc qPCR-R | GCCTCCAGCAGAAGGTGATCCA |
| CDC20 qPCR-F | ATCTGGAATGTGTGCTCTGG |
| CDC20 qPCR-R | CCTGAGATGAGCTCCTTGTAATG |
| CDC45 qPCR-F | GTCTTTGCCACCATGTCTTTG |
| CDC45 qPCR-R | GTACAGCTTGTCCAGGTTACTC |
| CCNA2 qPCR-F | TCACCAGACCTACCTCAAA |
| CCNA2 qPCR-R | GAGGAGAGAAACACCATGATAC |

**Table S5. Oligonucleotides used for inducible shRNA plasmid construction**

| Oligo | Sequence (5’-3’) |
|---|---|
| sh-IDH3α 1 top | CTAGCGGTGGTGTTCAGACAGTAATTCAAGAGATTACTGTCTG AACACCACCTTTTTG |
| sh-IDH3α 1 bottom | AATTCAAAAAGGTGGTGTTCAGACAGTAATCTCTTGAATTACTG TCTGAACACCACCG |
| sh-IDH3α 2 top | CTAGCGCTAAAGAGTCCATGGATATACTAGTTATCCATGGACTC TTTAGCTTTTTG |
| sh-IDH3α 2 bottom | AATTCAAAAAGCTAAAGAGTCCATGGATAACTAGTATATCCAT GGACTCTTTAGCG |
| sh-c-Myc 1 top | CTAGCCCTGAGACAGATCAGCAACAACTACTAGTGTTGTTGCT GATCTGTCTCAGGTTTTTG |
| sh-c-Myc 1 bottom | AATTCAAAAACCTGAGACAGATCAGCAACAACACTAGTAGTTG TTGCTGATCTGTCTCAGGG |
| sh-c-Myc 2 top | CTAGCGGACTATCCTGCTGCCAAGTACTAGTCTTGGCAGCAGG ATAGTCCTTTTTG |
| sh-c-Myc 2 bottom | AATTCAAAAAGGACTATCCTGCTGCCAAGACTAGTACTTGGCA GCAGGATAGTCCG |

**Table S6. Oligonucleotides used for ChIP**

| Oligo | Sequence (5'-3') |
|---|---|
| NDUFB7-ChIP-F | CCTAAATGACCCGGAAGCTG |
| NDUFB7-ChIP-R | CGAGGGTCACCTAGCTCCTAC |
| COX5B-ChIP-F | CTCCTGTCTCTGCAGCTTGTTCC |
| COX5B-ChIP-R | CCGCGAAGTAACCTTGAAGCC |
| UQCR10-ChIP-F | TCCCATAAAGTGTTGCACGTC |
| UQCR10-ChIP-R | CGCCATGTTTCTTCACAGTCCA |
| CCNA2-ChIP-F | CCAAAGAATAGTCGTAGCCGCCG |
| CCNA2-ChIP-R | TCGAGCTGGGTGAGCGAGC |
| CDC20-ChIP-F | CGGTTGGATTTTGAAGGAGCC |
| CDC20-ChIP-R | CAGTTCCGACCGGCTTTAAC |
| CDC45-ChIP-F | CGGGCGGAACTAAGCTACAA |
| CDC45-ChIP-R | CTGGGAACCCTCAGGGAAAG |
| IDH3A-ChIP-F | TGTCTTTCTCCACGGGATTG |
| IDH3A-ChIP-R | GACGTTTGTTCCACACCTACTA |
| IDH3B-ChIP-F | GCACTTTGTCTCTTGCTTTG |
| IDH3B-ChIP-R | TCAGGTCCTCGTCTGATAAA |
| IDH3G-ChIP-F | GTCAGTGCTGCCAGAGA |
| IDH3G-ChIP-R | AAGAGCGACGCGTAAGT |

**Table S7. Oligonucleotides used for luciferase reporter construction**

| Oligo | Sequence (5’-3’) |
|---|---|
| IDH3A promoter -HAF | GCTCGAGATCTGCGATCTAAGAGTCTCTGTTCCAGAAACAACGTCA |
| IDH3A promoter -HAR | TTTACCAACAGTACCGGAATGGAGGCTCCCGTGACCTTCCC |
| IDH3B promoter -HAF | GCTCGAGATCTGCGATCT TCCTGTACCAGTTTGTCTTTAGG |
| IDH3B promoter -HAR | TTTACCAACAGTACCGGACAGTTGACTTTGCCCTGTTTAG |
| IDH3G promoter -HAF | GCTCGAGATCTGCGATCTCCTTGCCTTAGCCTTCTCCATAAC |
| IDH3G promoter -HAR | TTTACCAACAGTACCGGAGTGGCTCTTTCCCTGCTCAC |
| CCNA2 promoter -HAF | GCTCGAGATCTGCGATCTGCTGTAACCAGTGGAGCTATT |
| CCNA2 promoter -HAR | TTTACCAACAGTACCGGACACTCTTCTCTGCCTGCTAAG |
| CDC20 promoter -HAF | GCTCGAGATCTGCGATCTGATGGTCTCGATCTTCTGACTTC |
| CDC20 promoter -HAR | TTTACCAACAGTACCGGAGTGCAGGTCACTCTCGAAC |
| CDC45 promoter -HAF | GCTCGAGATCTGCGATCTTCAACTCTCCAGTCCCTACTT |
| CDC45 promoter -HAR | TTTACCAACAGTACCGGATCAAGACTCCCGCCAAATC |

**Table S8. GSEA report for MYC targets genes**

| Name | Size | ES | NES | p-value | FDR, q-value | FWER, p |
|---|---|---|---|---|---|---|
| HALLMARK_MYC_TARGETS_V1 | 195 | 0.197687 | 0.864773 | 0.774446 | 0.816426 | 1 |
| HALLMARK_MYC_TARGETS_V2 | 58 | 0.288255 | 1.045651 | 0.395385 | 0.42958 | 1 |
